# Risk factors for vasovagal reactions in blood donors: A systematic review and meta‐analysis

**DOI:** 10.1111/trf.18078

**Published:** 2024-11-26

**Authors:** Yaning Wu, Hongchao Qi, Emanuele Di Angelantonio, Stephen Kaptoge, Angela M. Wood, Lois G. Kim

**Affiliations:** ^1^ British Heart Foundation Cardiovascular Epidemiology Unit, Department of Public Health and Primary Care University of Cambridge Cambridge UK; ^2^ Victor Phillip Dahdaleh Heart and Lung Research Institute University of Cambridge Cambridge UK; ^3^ National Institute for Health and Care Research Blood and Transplant Research Unit in Donor Health and Behaviour University of Cambridge Cambridge UK; ^4^ British Heart Foundation Centre of Research Excellence University of Cambridge Cambridge UK; ^5^ Health Data Research UK Cambridge Wellcome Genome Campus and University of Cambridge Cambridge UK; ^6^ Health Data Science Research Centre, Human Technopole Milan Italy; ^7^ Cambridge Centre of Artificial Intelligence in Medicine Cambridge United Kingdom; ^8^ British Heart Foundation Data Science Centre Health Data Research UK London UK

**Keywords:** donors

## Abstract

**Background:**

While blood donation is generally safe, some donors experience vasovagal reactions (VVRs) that may lead to injury and reduce likelihood of future donation. Several risk factors for VVRs have been identified, but the consistency, magnitude, and validity of their associations have not been systematically evaluated. Therefore, this systematic review and meta‐analysis synthesized evidence for VVR risk factors.

**Methods:**

Database searches identified English‐language studies published before February 2024 describing VVR risk factors in voluntary whole blood donors. Study characteristics, VVR and risk factor assessment methods, and effect sizes were extracted. Random‐effects models pooled estimates across all studies and subgroups of geographical context, study quality, donation experience, and outcome severity. Inconsistently and infrequently reported risk factors were narratively synthesized.

**Results:**

Totally 71 studies reporting a total of 19 million total donations were included. Female sex, new donor status, younger age, smaller blood volume, and lower blood pressure were positively associated with higher VVR risk. Donation‐related fear, anxiety, and disgust were associated with higher VVR risk in narrative syntheses. Substantial between‐study heterogeneity (*I* ^2^ > 90%) was observed for the majority of risk factors, while there was no clear evidence of subgroup variability and small study effects.

**Conclusion:**

This systematic review and meta‐analysis provides a comprehensive synthesis of risk factors for VVRs across wide‐ranging blood service contexts and symptom severities, reinforcing evidence for previously identified factors. The heterogeneous associations of several risk factors motivate large‐scale studies that enable comprehensive multivariable adjustment to evidence donor selection criteria and preventative intervention allocation.

**Abbreviations**
BPM: Beats per minute
CI: Confidence interval
DBP: Diastolic blood pressure
EBV: Estimated blood volume
GL: Greenland and Longnecker
ISBT: International Society of Blood Transfusion
NOS: Newcastle‐Ottawa Scale
OR: Odds ratio
PRISMA: Preferred Reporting Items for Systematic Reviews and Meta‐Analyses
PROSPERO: The International Prospective Register of Systematic Reviews
RCS: Restricted cubic spline
RCT: Randomized controlled trial
RR: Risk ratio
SBP: Systolic blood pressure
VVR: Vasovagal reaction

## 1 INTRODUCTION

Whole blood and its constituent products, designated as essential drugs by the World Health Organization,^1^ are used to treat a wide range of chronic and acute conditions. Supplies of blood and blood products must be maintained by regular donations from healthy individuals, which often constitutes voluntary, non‐renumerated donation in most high‐income countries.^2^ Though blood donation is considered safe, some donors suffer adverse events such as vasovagal reactions (VVRs), which present as faintness and dizziness with or without actual loss of consciousness (LOC). VVRs with LOC (or syncope) occur during or after 0.1–0.5% of whole blood donations worldwide, while VVRs without LOC (or presyncope) are more common, with a worldwide prevalence of 1.4%–7%.^3^

Though most VVRs are self‐limiting, a small proportion of affected donors suffer traumatic injuries necessitating healthcare intervention,^4^, ^5^ and even mild reactions can reduce the likelihood of future donations.^6^, ^7^, ^8^ In view of their relevance to donor health, experience, and retention, advanced understanding of risk factors for VVRs is necessary to intervene on this complication.

Several observational studies published to date have examined risk factors for VVRs. A 2019 systematic review focused exclusively on predictors of syncopal (i.e., LOC‐associated) VVRs related to donation across 11 studies,^9^ while an earlier narrative review described risk factors for both presyncopal (i.e., non‐LOC‐associated) and syncopal VVRs.^10^ These studies identified demographic, anthropometric, biomarker, donation‐specific, and psychologic factors associated with VVRs.^9^, ^10^ Furthermore, other reviews have identified low‐quality evidence describing null associations between donation complications (including, but not limited to, VVRs) and epilepsy,^11^ pre‐donation hypotension,^12^ and treated hypertension and non‐insulin‐dependent diabetes mellitus.^13^ Despite accumulating literature on risk factors for VVRs, no comprehensive quantitative synthesis on this topic has ever been conducted, and individual study quality has never been systematically evaluated.

As blood services attempt to recruit and retain donors in response to diversifying clinical demands,^14^ averting both presyncopal and syncopal VVRs will be central to protecting donor health and stewarding blood supplies. To match growing global interest in preventative strategies for these complications,^15^ a comprehensive understanding of high‐risk donor profiles is needed to inform global donor selection practices and target preventative interventions. Therefore, we conducted a systematic review and meta‐analysis to evaluate risk factors for VVRs in voluntary whole blood donors.

## 2 METHODS

This systematic review and meta‐analysis was registered prospectively on PROSPERO (identifier CRD42023476467) and followed the Preferred Reporting Items for Systematic Reviews and Meta‐Analyses (PRISMA) 2020 guidelines^16^ (Appendix S1).

### 2.1 Search strategy and selection criteria

We searched MEDLINE, Embase, Web of Science Core Collections, the Cochrane Library, and APA PsycINFO to identify primary studies of risk factors for presyncopal or syncopal VVRs (as defined by International Society for Blood Transfusion [ISBT] guidelines^17^) during or after voluntary whole blood donation published from database inception until February 1st, 2024. In addition, all articles from the Transfusion Evidence Library under the Subject Areas of “Donor Care” and “Recruitment and Retention of Blood Donors” were extracted and screened due to the platform's lack of comprehensive search functionality (search terms detailed in Appendix S2). Reference lists of identified reviews were also hand‐searched for relevant studies.

Article titles and abstracts were initially screened for relevance by two independent reviewers, HQ (10% of articles) and YW (100% of articles). Full texts were similarly screened for eligibility by both reviewers, with conflicts resolved by discussion. Inclusion and exclusion criteria are detailed in Appendix S3.

### 2.2 Data extraction and quality assessment

YW and HQ independently extracted data from a random 10% of eligible articles using a standardized pilot form, collecting information about study and participant characteristics, data sources, outcome definitions, and risk factors. The relevance and consistency of extracted data were checked by both reviewers with disagreements resolved by discussion. YW then extracted data from the remaining 90% of articles. Data were only extracted from publications describing distinct study populations except where multiple publications described different risk factors within the same study population.

Unadjusted and adjusted odds ratios (ORs) and their 95% confidence intervals (CIs) were extracted where available. For studies describing the number of VVRs occurring across risk factor groups, ORs and 95% CIs were calculated. When associations were only reported separately by subgroups (e.g., male and female donors), they were pooled using fixed effects meta‐analysis to obtain a single estimate per study. For studies displaying ORs and 95% CIs only in plots, without numeric values,^18^, ^19^, ^20^, ^21^ an online tool (https://eleif.net/photomeasure)^22^ was used to derive approximate numeric values. ORs are henceforth interpreted as risk ratios (RRs) given the low incidence of VVRs.^3^

We used revised Newcastle‐Ottawa Scales^23^ (NOS) to assess studies' risk of bias. Our bespoke revisions (Appendix S4) ensured that items could distinguish between included studies of higher and lower quality given the unique design considerations characterizing voluntary blood donation research, such as the use of only “healthy” community controls in all case–control studies.

### 2.3 Data analysis

We used random‐effects meta‐analysis models^24^ to pool standardized estimates of risk factor associations with VVRs due to substantial heterogeneity related to differing populations, donation procedures, and study designs.

Where binary risk factors were defined and reported similarly across three or more studies, unadjusted and adjusted estimates were pooled separately using DerSimonian and Laird's method^25^ with standard errors and significance tests based on Hartung‐Knapp‐Sidik‐Jonkman corrections to mitigate substantial heterogeneity.^26^

Associations of continuous risk factors described across three or more studies were pooled after transforming them to a common scale of comparison across studies using previously published methods (detailed in Appendix S5).^27^ In brief, RRs and 95% CIs comparing risk factor values above and below a common cutoff value were converted to RRs and 95% CIs comparing highest and lowest thirds of risk factor distributions while assuming normality and log‐linear associations.

Due to assumed non‐normality of age and potential nonlinear associations with VVR,^28^ dose–response log‐linear associations per one‐year higher age were modeled across the age distributions using a two‐stage model proposed by Greenland and Longnecker (GL)^29^ and Orsini^30^ using methods detailed in Appendix S6.

We assessed heterogeneity using the *I* ^2^ statistic^31^ and explored risk factors whose pooled estimates exhibited substantial between‐study heterogeneity (*I* ^2^ ≥ 50%)^32^ using prespecified subgroup analyses. In these analyses, unadjusted risk factor RRs and 95% CIs were pooled separately across (1) studies or participant subsets reporting presyncopal versus syncopal VVR outcomes, (2) studies or subsets including only new versus only returning donors, (3) studies conducted in Western versus non‐Western settings, and (4) studies with higher than/equal to versus lower than median total NOS scores. Analyses were only conducted when ≥3 studies or study subsets were available per subgroup.

Small study effects, including publication bias, were assessed by funnel plots^33^ and Thompson arcsine tests^34^ for risk factors described by ≥10 studies.^32^ Prespecified sensitivity analyses explored the robustness of pooled estimates to reported missing data (methods detailed in Appendix S7). Where risk factor definitions were insufficiently consistent to justify meta‐analysis (i.e., due to differing scales), associations were described using narrative synthesis.

All analyses were conducted using R Statistical Software (version 4.3.2, R Core Team).

## 3 RESULTS

**FIGURE 1:**
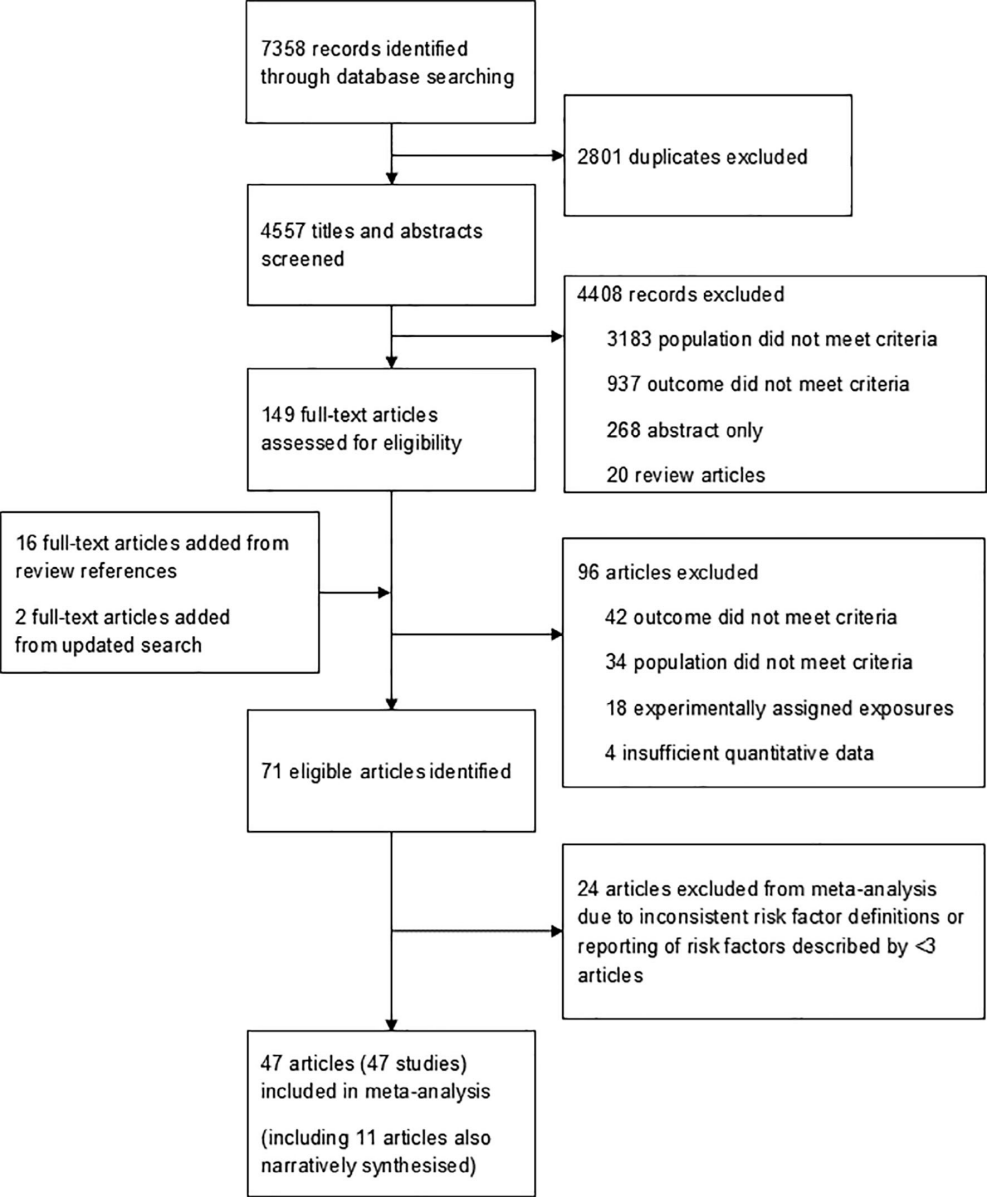
Flow diagram of study selection.

Of 167 full texts screened, we identified 71 studies fulfilling inclusion criteria (Figure 1). Totally 47 studies including 18,892,752 total donations reported sufficient summary data for quantitative synthesis of at least one risk factor and 35 studies including 1,945,470 donations were narratively synthesized, including 11 studies that were also quantitatively synthesized.

### 3.1 Study characteristics and quality

Of the 71 studies, seven were conducted primarily (≥80%) on male donors,^35^, ^36^, ^37^, ^38^, ^39^, ^40^, ^41^ 22 on young (≤25 years) donors,^19^, ^21^, ^41^, ^42^, ^43^, ^44^, ^45^, ^46^, ^47^, ^48^, ^49^, ^50^, ^51^, ^52^, ^53^, ^54^, ^55^, ^56^, ^57^, ^58^, ^59^, ^60^, ^61^ and 55 in Western population settings.^5^, ^8^, ^18^, ^19^, ^20^, ^21^, ^37^, ^42^, ^43^, ^44^, ^45^, ^46^, ^48^, ^50^, ^51^, ^52^, ^53^, ^54^, ^55^, ^56^, ^57^, ^58^, ^59^, ^60^, ^61^, ^62^, ^63^, ^64^, ^65^, ^66^, ^67^, ^68^, ^69^, ^70^, ^71^, ^72^, ^73^, ^74^, ^75^, ^76^, ^77^, ^78^, ^79^, ^80^, ^81^, ^82^, ^83^, ^84^, ^85^, ^86^, ^87^, ^88^, ^89^, ^90^, ^91^, ^92^, ^93^ Three studies recorded only syncopal VVRs,^18^, ^37^, ^94^ while the remaining studies recorded both syncopal and presyncopal VVRs (Table 1 and Appendix S8). Totally 44 studies (62%) did not adjust for any covariates; hence, the present study focused primarily on meta‐analyses of unadjusted estimates. Outcome ascertainment was generally high quality, with standardized recording of VVRs by a phlebotomist used in 55 (77%) studies. Only 41 (58%) studies reported no missing risk factor and outcome data. Cross‐sectional studies included less population‐representative samples than case–control and cohort studies, and quantitatively synthesized studies had higher NOS scores than narratively synthesized studies on average (Appendix S9).

**TABLE 1:** Descriptive statistics of included studies.

	Meta‐analyzed studies (*N* = 47)	Narratively synthesized studies (*N* = 24)^a^
Western context (*N*, %)	33 (70)	24 (100)
Number of included donations (*N*, %)		
1–1000 donations	9 (19)	17 (71)
1001–10,000 donations	11 (23)	5 (21)
10,001–100,000 donations	12 (26)	0 (0)
100,001‐1000,000 donations	12 (26)	2 (8)
>1000,001 donations	3 (6)	0 (0)
VVR incidence^b^ (median, IQR)	3 (7)	12 (8)
% female participants^c^ (median, IQR)	48 (18)	53 (11)
Use of phlebotomist‐assessed VVRs (*N*, %)	41 (87)	15 (62)
Inclusion of only syncopal VVRs (*N*, %)	3 (6)	0 (0)

Abbreviation: IQR, interquartile range.

^a^ Excludes 11 studies included in both quantitative and narrative syntheses.

^b^ 1 meta‐analyzed study and 13 narratively synthesized studies did not report VVR incidence (including those reporting only continuous scores on the Blood Donation Reactions Inventory (BDRI; see France et al), which has no validated cutoff to determine whether a VVR has occurred).

^c^ Five meta‐analyzed studies and two narratively synthesized studies did not report % female participants.

### 3.2 Quantitative syntheses

**FIGURE 2:**
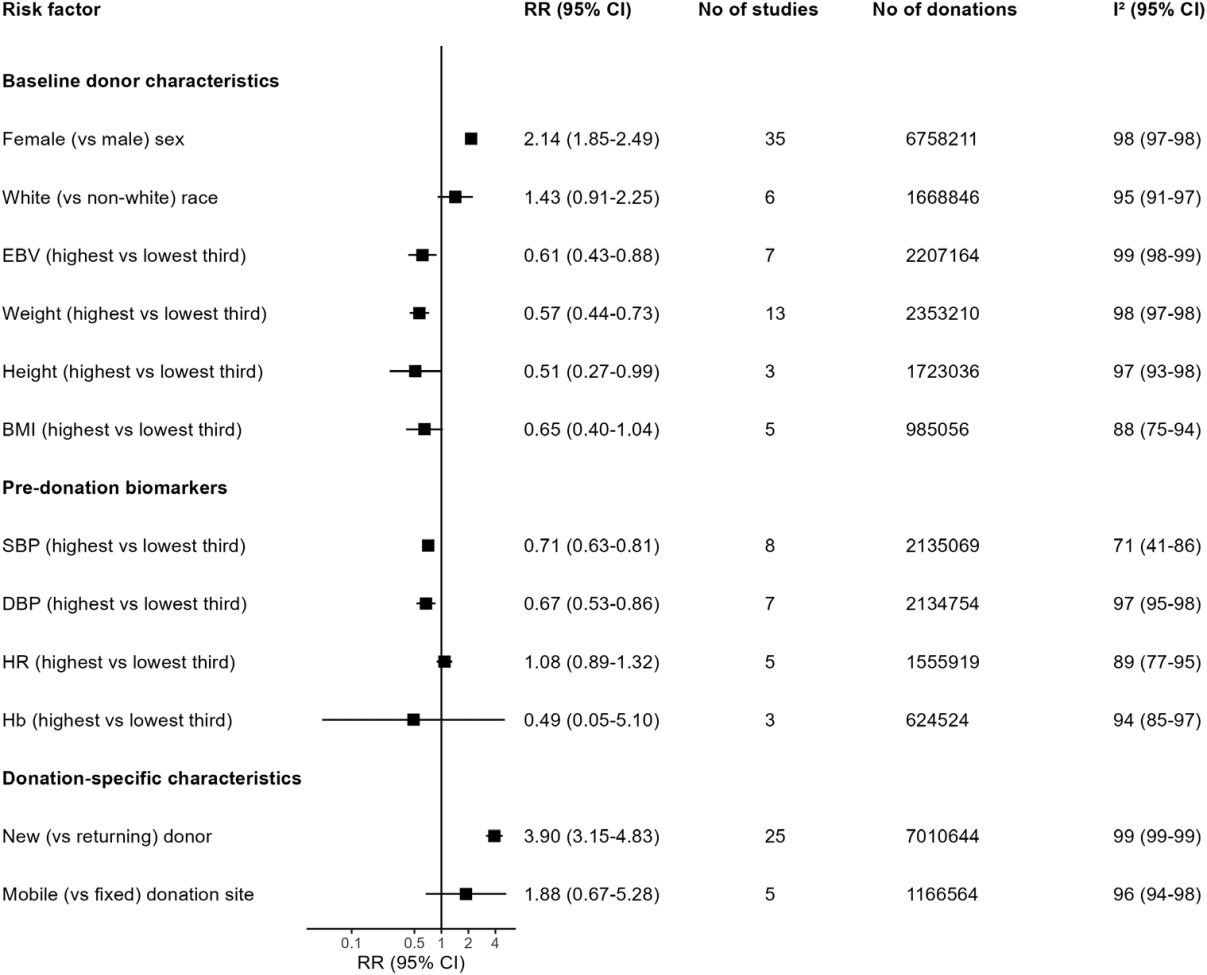
Unadjusted associations for all meta‐analyzed risk factors other than age. EBV: estimated blood volume. BMI: body mass index. SBP: systolic blood pressure. DBP: diastolic blood pressure. HR: heart rate. Hb: hemoglobin. RR: risk ratio. CI: confidence interval.

**FIGURE 3:**
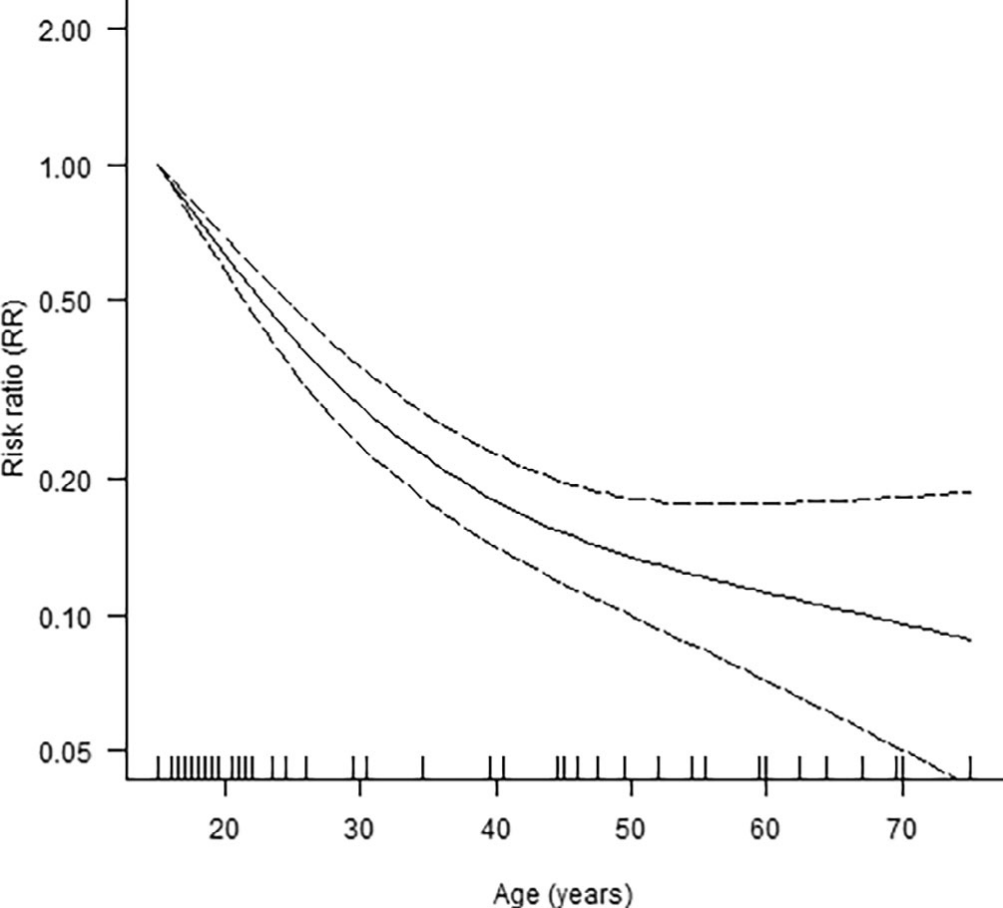
Unadjusted non‐log‐linear dose–response association between age and VVRs (k = 15 studies; *n* = 93,600 donations; *p*‐nonlinearity <0.001; per‐year log‐linear dose–response RR: 0.95; 95% CI: 0.94, 0.96; *I* ^2^: 98%; 95% CI: 98%, 99%). The solid line represents the dose–response curve, while dashed lines represent the corresponding 95% confidence interval.

The main findings for quantitatively synthesized risk factor associations are displayed in Figures 2 and 3.

#### 3.2.1 Baseline donor characteristics

Across 35 studies of nearly seven million donations, female donors had 2.14 times (95% CI: 1.82, 2.49; *I* ^2^: 98%) higher risk of VVRs than male donors. Moreover, across 15 studies, every one‐year higher donor age was, on average, associated with 0.95 times (95% CI: 0.94, 0.96; *I* ^2^: 98%) the risk of VVRs across 15 studies, although dose–response meta‐analysis suggested a potentially non‐log‐linear association that plateaued after age 50 years (Figure 3). In addition, across five US studies^8^, ^18^, ^19^, ^73^, ^84^ and one South African study^47^ examining race or ethnicity, there was limited evidence of higher VVR risk in white donors compared with all non‐white donors (RR: 1.43; 95% CI: 0.91, 2.25; *I* ^2^: 95%). Notably, five studies comparing black and white donors^18^, ^19^, ^47^, ^73^, ^84^ reported substantially lower VVR risk in black donors compared with white donors, even after adjustment for demographic and donation‐specific covariates.

Across seven studies, higher estimated blood volume (EBV; calculated as a function of donor sex, weight, and height^95^) was associated with lower VVR risk, with donors in the top third of EBV distributions having 0.61 times (95% CI: 0.43, 0.88; *I* ^2^: 99%) lower VVR risk compared with those in the bottom third. Weight, height, and BMI showed pooled estimates identical in direction and similar in magnitude to EBV, though limited evidence for associations with BMI were found.

Adjustment for various demographic and anthropometric characteristics moderately attenuated the pooled association estimates for sex and EBV (Appendix S10).

#### 3.2.2 Pre‐donation biomarkers

Associations of pre‐donation SBP and DBP with VVRs were described by eight and seven studies respectively that included around two million donations each. Donors in the highest thirds of SBP and DBP distributions had 0.71 times (95% CI: 0.63, 0.80; *I* ^2^: 71%) and 0.67 times (95% CI: 0.53, 0.86; *I* ^2^: 97%) lower VVR risk respectively compared with those in the lowest thirds.

Furthermore, across five studies, faster heart rate (HR) showed no evidence of an association with VVRs when comparing donors in the highest and lowest thirds of HR distributions (RR: 1.08; 95% CI: 0.89, 1.32; *I* ^2^: 89%). Similarly, pre‐donation hemoglobin showed no evidence for an association with VVR across three studies when comparing donors in the highest and lowest thirds of distributions (RR: 0.49; 95% CI: 0.05, 5.10; *I* ^2^: 94%).

#### 3.2.3 Donation‐specific factors

Twenty‐four studies of seven million donations found that new donors had 3.86 times (95% CI: 3.09, 4.82; *I* ^2^: 99%) the VVR risk compared with returning donors. This estimate was attenuated by one‐third when adjusted for diverse covariates (Figure S1). In addition, no evidence was found for an association between donation site type (i.e., mobile versus fixed site) with VVRs in five studies (RR: 1.88; 95% CI: 0.67, 5.28; *I* ^2^: 96%).

### 3.3 Narrative synthesis

Findings from narrative syntheses of risk factors for VVRs are detailed in Appendix S11. In brief, numbers of previous donations, VVR history, seeing another VVR, poor phlebotomist interpersonal skills, longer blood draw time, biomarker fluctuations during donation, donation‐related fear and negative affect, any fainting history, and pre‐donation hunger, thirst, and both short (≤5 or 6 hours) and long (≥9 hours) sleep were associated with higher VVR risk across single or multiple studies. Moreover, anxiety and medical history had inconsistent associations across multiple studies, while none showed evidence that weather season was associated with VVR risk. Notably, associations for fear and anxiety were generally consistent within studies assessing both phlebotomist‐recorded and donor‐recalled VVRs.^51^, ^52^, ^53^, ^54^, ^58^, ^59^, ^92^

### 3.4 Subgroup and sensitivity analyses

Analyses stratified by syncopal versus presyncopal outcomes, new versus returning donor status, Western versus non‐Western contexts, and higher‐than/equal to versus lower‐than‐median study quality produced estimates with similar point estimates (Appendix S12). Notably, however, positive associations with female sex appeared stronger in returning donors and in lower quality studies, though no formal statistical tests were conducted given the low numbers of studies available. In addition, negative dose–response associations with older age were weaker in new donors and negative associations with higher weight were stronger for syncopal outcomes. Furthermore, sensitivity analyses using imputed data for three studies with risk factor missingness^18^, ^19^, ^96^ produced negligible differences from original estimates (Appendix S13).

### 3.5 Small study effects

Funnel plots for sex, age, weight, and donation experience were generally symmetric, though unusually, less heterogeneity was generally observed in smaller studies than larger studies (Appendix S14). Thompson's arcsine tests produced p‐values >0.05 for all risk factors other than weight (*p* = 0.01), indicating that effect sizes and precision were likely unrelated, though power was limited.^34^

## 4 DISCUSSION

### 4.1 Summary of findings

Our systematic review and meta‐analysis integrated data from 71 studies across five decades of donation safety research to produce the most comprehensive quantitative synthesis on risk factors for donation‐related VVRs to date. Across diverse outcome severities and blood service contexts, we found positive associations of female sex, new donor status, younger age, and lower EBV, weight, height, SBP, and DBP with higher VVR risk. Substantial heterogeneity (*I* ^2^ > 90%) was observed across all risk factors, though differences in outcome severity, donation experience, geographic context, and study quality did not appear to explain this heterogeneity. Quality assessments found a high standard of outcome ascertainment across included studies, though the majority lacked covariate adjustment and failed to describe data missingness. In light of these analytic characteristics, pooled estimates should be interpreted with caution.

### 4.2 Findings in context

Risk factors for donation‐related VVRs identified in this study, including female sex, younger age, and VVR history, have previously been reported in studies of lifetime VVRs with any trigger.^97^, ^98^ Similar risk factors have also been reported in studies of VVRs triggered by medical procedures such as vaccine administration^99^ and blood testing.^100^ Interestingly, two studies of VVRs in patient populations undergoing injections at lower body sites (i.e., for medication or analgesic administration or diagnostic testing) found no difference in risk by patient sex, age, weight, or blood pressure,^101^, ^102^ while another study in similar populations found higher risk in older men.^103^ This suggests that risk factors for donation‐related VVRs likely overlap with those for VVRs in generally healthy populations caused by everyday triggers, while they may differ from risk factors in patient populations.

This work's syntheses of predominantly unadjusted risk factor associations with VVRs are unable to infer causality. However, they suggest that individual‐level factors, including smaller body compositions and medical fears, affect donors' physiologic and psychologic responses to volume loss and needle exposure.^104^ Indeed, small trials have shown that pre‐donation hydration may prevent VVRs by compensating for absolute volume reduction.^105^, ^106^, ^107^ On the other hand, gastric distension may also contribute to this intervention's efficacy.^108^ Laboratory studies have shown, too, that female sex and younger age may impart additional vulnerability to VVRs due to physiologic differences in cardiovascular reactivity in these groups.^109^, ^110^, ^111^ Moreover, lower pre‐donation blood pressure in VVR‐affected donors may result from a baseline “hypotensive phenotype” that predisposes individuals to orthostatic intolerance.^112^ Alternatively, this transient hypotension could, itself, signal the beginning of vasovagal reflex activation.^113^ Importantly, demographic, anthropometric, and psychologic characteristics associated with higher VVR incidence may present simultaneously in many affected donors.^114^, ^115^, ^116^, ^117^

Findings of associations of VVRs with donation‐specific factors such as donation history, blood draw fears, and phlebotomist skill suggest that prevention strategies should be tailored to this setting and population. Notably, strong positive associations of VVRs with few prior donation experiences and negative prior experiences (i.e., previous VVRs) motivate further investigations of how, and why, donor vulnerability may change with accumulating experience. These findings also suggest the need for enhanced blood service vigilance in inexperienced and previously reacting donors, as already recommended by some national guidelines.^118^ Preventing VVRs and stewarding donor health requires evidence‐based approaches to donor selection, monitoring, and communications.

### 4.3 Strengths and limitations

Our study has key strengths. First, it presents the most comprehensive quantitative synthesis of VVR risk factors to date, adding new global data to previously published systematic^9^ and narrative^10^ reviews that included studies from predominantly Western contexts. In addition, our meta‐analyses quantified associations for various continuous risk factors by harmonizing study estimates. The veracity of our assumptions of log‐linearity between these factors and VVRs are supported by dose–response estimates from large, population‐representative studies.^18^, ^21^ Our synthesis also provided detailed evaluations of the quality of included studies, illuminating gaps in covariate adjustment and missing data reporting that can be addressed by future research on VVRs.

However, several limitations are also salient. First, despite our broad search strategy, this review may have excluded relevant evidence due to the preferential publishing of English‐language studies from the Global North with positive findings.^119^, ^120^ Relatedly, we were unable to include studies that reported summary outcome data across whole blood and apheresis or replacement and voluntary donors combined,^121^, ^122^, ^123^, ^124^, ^125^, ^126^ and more work is required to elucidate potential differences in VVR risk factors between these groups. Indeed, this synthesis' exclusion of replacement donors disproportionately excludes potentially relevant studies from low‐ and middle‐income settings within which these donors are predominantly based.^127^ This exclusion, as well as this review's English‐language inclusion criterion, may have limited its findings' generalizability to these lower‐resource contexts. For example, pooled associations for donation site type may have failed to capture cross‐context variation in differences between fixed and mobile sites' environments. Second, the associations synthesized in this study are unable to infer causality. In particular, the pooled unadjusted associations produced by our syntheses are likely biased due to their inability to account for confounding, but these findings reflect the associations reported by included studies. Future syntheses may use individual participant data to examine mutually adjusted risk factor associations. These syntheses may also facilitate statistical testing of subgroup differences where insufficient power was available for these tests in the present study. Third, our syntheses used donations, not donors, as units of analysis, meaning that returning donors may have contributed repeated risk factor measurements, especially in large, population‐wide studies. Prospective cohorts that follow donors throughout multiple donation instances while accounting for risk factor clustering are therefore necessary. Fourth, the substantial heterogeneity (*I* ^2^ > 90%) observed for most meta‐analyzed risk factors indicate that pooled estimates can only be considered averages of associations across contexts. This heterogeneity may be attributed to differences in participant and donor characteristics, donation procedures, and/or outcome operationalization across studies, donation settings, and time. Notably, the vast majority of study‐specific risk factor associations were consistent in direction, offering some reassurance that conclusions based on our analyses are broadly appropriate. Finally, this synthesis excluded studies of genetic risk factors for VVRs despite their potential relevance to risk prediction, though only one genome‐wide association study of donation‐related VVRs in specific has been published to date.^128^

## 5 CONCLUSION

In sum, diverse individual and contextual characteristics, including potentially modifiable factors, are associated with higher risk of VVRs during or after whole blood donation. Our findings motivate future analyses using comprehensive multivariable adjustment and accounting for genetic determinants to disentangle the complex etiology of VVRs. Building evidence in this area can support the identification of at‐risk donors and the development and allocation of VVR interventions. Efforts to prevent these complications can steward donor health and retention and ensure safe and sustainable blood supplies across the globe.

## CONFLICT OF INTEREST STATEMENT

The authors declare no conflict of interest.

## Supporting information


**Data S1.** Supporting Information.
