# Supplementary material for: Risk factors for vasovagal reactions in blood donors: A systematic review and meta‐analysis
Source: Transfusion. 2024 Nov 26;65(1):211–23. doi: 10.1111/trf.18078 (PMC11747113; doi:10.1111/trf.18078)
Supplement: Supplementary file 1 — Data S1. Supporting Information. [file TRF-65-211-s001.docx]

**SUPPLEMENTARY DATA**

**Appendix S1**: PRISMA checklist

| **Section/topic** | **#** | **Checklist item** | **Reported in section** |
| --- | --- | --- | --- |
| **TITLE** |  | | |
| Title | 1 | Identify the report as a systematic review, meta-analysis, or both. | Title |
| **ABSTRACT** |  | | |
| Structured summary | 2 | Provide a structured summary including, as applicable: background; objectives; data sources; study eligibility criteria, participants, and interventions; study appraisal and synthesis methods; results; limitations; conclusions and implications of key findings; systematic review registration number. | N/A |
| **INTRODUCTION** |  | | |
| Rationale | 3 | Describe the rationale for the review in the context of what is already known. | Introduction |
| Objectives | 4 | Provide an explicit statement of questions being addressed with reference to participants, interventions, comparisons, outcomes, and study design (PICOS). | Introduction |
| **METHODS** |  | | |
| Protocol and registration | 5 | Indicate if a review protocol exists, if and where it can be accessed (e.g., Web address), and, if available, provide registration information including registration number. | Methods |
| Eligibility criteria | 6 | Specify study characteristics (e.g., PICOS, length of follow-up) and report characteristics (e.g., years considered, language, publication status) used as criteria for eligibility, giving rationale. | Methods and supplementary data |
| Information sources | 7 | Describe all information sources (e.g., databases with dates of coverage, contact with study authors to identify additional studies) in the search and date last searched. | Methods and supplementary data |
| Search | 8 | Present full electronic search strategy for at least one database, including any limits used, such that it could be repeated. | Supplementary data |
| Study selection | 9 | State the process for selecting studies (i.e., screening, eligibility, included in systematic review, and, if applicable, included in the meta-analysis). | Methods |
| Data collection process | 10 | Describe method of data extraction from reports (e.g., piloted forms, independently, in duplicate) and any processes for obtaining and confirming data from investigators. | Methods |
| Data items | 11 | List and define all variables for which data were sought (e.g., PICOS, funding sources) and any assumptions and simplifications made. | Methods |
| Risk of bias in individual studies | 12 | Describe methods used for assessing risk of bias of individual studies (including specification of whether this was done at the study or outcome level), and how this information is to be used in any data synthesis. | Methods and supplementary data |
| Summary measures | 13 | State the principal summary measures (e.g., risk ratio, difference in means). | Methods |
| Synthesis of results | 14 | Describe the methods of handling data and combining results of studies, if done, including measures of consistency (e.g., I^2^) for each meta-analysis. | Methods and supplementary data |
| Risk of bias across studies | 15 | Specify any assessment of risk of bias that may affect the cumulative evidence (e.g., publication bias, selective reporting within studies). | Methods |
| Additional analyses | 16 | Describe methods of additional analyses (e.g., sensitivity or subgroup analyses, meta-regression), if done, indicating which were pre-specified. | Methods and supplementary data |
| **RESULTS** |  |  |  |
| Study selection | 17 | Give numbers of studies screened, assessed for eligibility, and included in the review, with reasons for exclusions at each stage, ideally with a flow diagram. | Results |
| Study characteristics | 18 | For each study, present characteristics for which data were extracted (e.g., study size, PICOS, follow-up period) and provide the citations. | Results and supplementary data |
| Risk of bias within studies | 19 | Present data on risk of bias of each study and, if available, any outcome level assessment (see item 12). | Results and supplementary data |
| Results of individual studies | 20 | For all outcomes considered (benefits or harms), present, for each study: (a) simple summary data for each intervention group (b) effect estimates and confidence intervals, ideally with a forest plot. | Results and supplementary data |
| Synthesis of results | 21 | Present results of each meta-analysis done, including confidence intervals and measures of consistency. | Results and supplementary data |
| Risk of bias across studies | 22 | Present results of any assessment of risk of bias across studies (see Item 15). | Results and supplementary data |
| Additional analysis | 23 | Give results of additional analyses, if done (e.g., sensitivity or subgroup analyses, meta-regression [see Item 16]). | Results and supplementary data |
| **DISCUSSION** |  |  |  |
| Summary of evidence | 24 | Summarize the main findings including the strength of evidence for each main outcome; consider their relevance to key groups (e.g., healthcare providers, users, and policy makers). | Discussion |
| Limitations | 25 | Discuss limitations at study and outcome level (e.g., risk of bias), and at review-level (e.g., incomplete retrieval of identified research, reporting bias). | Discussion |
| Conclusions | 26 | Provide a general interpretation of the results in the context of other evidence, and implications for future research. | Discussion |
| **FUNDING** |  |  |  |
| Funding | 27 | Describe sources of funding for the systematic review and other support (e.g., supply of data); role of funders for the systematic review. | Acknowledgements |

*Taken from*  Moher D, Liberati A, Tetzlaff J, Altman DG, The PRISMA Group (2009). Preferred Reporting Items for Systematic Reviews and Meta-Analyses: The PRISMA Statement. PLoS Med 6(7): e1000097. doi:10.1371/journal.pmed1000097

For more information, visit: **www.prisma-statement.org**.

**Appendix S2**: Systematic search strategy

| **Database** | **Terms** |
| --- | --- |
| MEDLINE via Ovid | From 1946    1 Blood Donors/ or Blood Donation/  2 (blood adj (donat* or donor* or drive*)).ab,kf,kw,ti.  3 1 or 2  4 "Correlation of Data"/ or Risk Factors/ or Epidemiology/  5 (correlat* or assoc* or risk* or predict* or factor* or epidemiolog*).ab,kf,kw,ti.  6 4 or 5  7 Syncope, Vasovagal/ or Syncope/ or Dizziness/ or Hypotension/ or Bradycardia/ or Sweat/ or Nausea/ or Vomiting/ or Pallor/  8 (vasovagal or syncop* or faint* or dizz* or lightheaded or hypotens* or bradycardi* or unconscious* or sweat* or nause* or vomit* or pallor or adverse or symptom*).ab,kf,kw,ti.  9 7 or 8  10 3 and 6 and 9 |
| EMBASE via Ovid | From 1974    1 blood donor/  2 (blood adj (donat* or donor* or drive*)).ab,kf,kw,ti.  3 1 or 2  4 risk factor/ or correlation analysis/ or epidemiology/ or prediction/  5 (correlat* or assoc* or risk* or predict* or factor* or epidemiolog*).ab,kf,kw,ti.  6 4 or 5  7 faintness/ or syncope/ or dizziness/ or hypotension/ or orthostatic hypotension/ or hemorrhagic hypotension/ or bradycardia/ or sweat/ or nausea/ or vomiting/ or pallor/ or adverse outcome/  8 (vasovagal or syncop* or faint* or dizz* or lightheaded or hypotens* or bradycardi* or unconscious* or sweat* or nause* or vomit* or pallor or adverse or symptom*).ab,kf,kw,ti.  9 7 or 8  10 3 and 6 and 9 |
| Web of Science Core Collections via Web of Science | From 1990  1 ((TS=((blood NEAR/1 (donat* OR donor* OR drive*)))  2 ((TS=((correlat* OR assoc* OR risk* OR predict* OR factor* OR epidemiolog*)))  3 1 and 2  4 ((TS=(vasovagal OR syncop* OR faint* OR dizz* OR lighthearted OR hypotens* OR bradycardi* OR unconscious* OR sweat* OR nause* OR vomit* OR pallor OR adverse OR symptom*)))  5 1 and 2 and 4 |
| Cochrane Library | From 1996 (Cochrane Database of Systematic Reviews)  From 1908 (CENTRAL Trials)  From 2012 (Cochrane Clinical Answers)  From 2010 (Cochrane Editorials)  #1 (blood NEAR/1 (donat* OR donor* OR drive*)):ti,ab,kw (Word variations have been searched)  #2 MeSH descriptor: [Blood Donation] explode all trees  #3 MeSH descriptor: [Blood Donors] explode all trees  #4 #1 OR #2 OR #3  #5 (correlat* OR assoc* OR risk* OR predict* OR factor* OR epidemiolog*):ti,ab,kw  #6 MeSH descriptor: [Correlation of Data] explode all trees  #7 MeSH descriptor: [Risk Factors] explode all trees  #8 MeSH descriptor: [Predictive Value of Tests] explode all trees  #9 MeSH descriptor: [Epidemiology] explode all trees  #10 #5 OR #6 OR #7 OR #8 OR #9  #11 (vasovagal OR syncop* OR faint* OR dizz* OR lightheaded OR hypotens* OR bradycardi* OR unconscious* OR sweat* OR nause* OR vomit* OR pallor OR adverse OR symptom*):ti,ab,kw  #12 MeSH descriptor: [Syncope, Vasovagal] explode all trees  #13 MeSH descriptor: [Syncope] explode all trees  #14 MeSH descriptor: [Dizziness] explode all trees  #15 MeSH descriptor: [Hypotension] explode all trees  #16 MeSH descriptor: [Bradycardia] explode all trees  #17 MeSH descriptor: [Unconsciousness] explode all trees  #18 MeSH descriptor: [Sweating] explode all trees  #19 MeSH descriptor: [Nausea] explode all trees  #20 MeSH descriptor: [Vomiting] explode all trees  #21 MeSH descriptor: [Pallor] explode all trees  #22 #11 OR #12 OR #13 OR #14 OR #15 OR #16 OR #17 OR #18 OR #19 OR #20 OR #21  #23 #4 AND #10 AND #22 |
| APA PsychInfo via ProQuest | From 1894  Set#: S1  Searched for: tiab((blood NEAR/1 (donat* OR donor* OR drive*)))  Databases: APA PsycInfo®    Set#: S2  Searched for: tiab(correlat* OR assoc* OR risk* OR predict* OR factor* OR epidemiolog*)  Databases: APA PsycInfo®    Set#: S3  Searched for: MAINSUBJECT.EXACT("Predictive Analysis") OR MAINSUBJECT.EXACT("Statistical Correlation") OR MAINSUBJECT.EXACT("Epidemiology")  Databases: APA PsycInfo®    Set#: S4  Searched for: [S2] OR [S3]  Databases: APA PsycInfo®  These databases are searched for part of your query.    Set#: S5  Searched for: tiab(vasovagal OR syncop* OR faint* OR dizz* OR lightheaded OR hypotens* OR bradycardi* OR unconscious* OR sweat* OR nause* OR vomit* OR pallor OR adverse OR symptom*)  Databases: APA PsycInfo®    Set#: S6  Searched for: MAINSUBJECT.EXACT("Syncope") OR MAINSUBJECT.EXACT("Vertigo") OR MAINSUBJECT.EXACT("Hypotension") OR MAINSUBJECT.EXACT("Bradycardia") OR MAINSUBJECT.EXACT("Unconsciousness") OR MAINSUBJECT.EXACT("Sweating") OR MAINSUBJECT.EXACT("Nausea") OR MAINSUBJECT.EXACT("Vomiting") OR MAINSUBJECT.EXACT("Symptoms")  Databases: APA PsycInfo®    Set#: S7  Searched for: [S5] OR [S6]  Databases: APA PsycInfo®  These databases are searched for part of your query.  Set#: S8  Searched for: [S1] AND [S4] AND [S7]  Databases: APA PsycInfo®  These databases are searched for part of your query. |
| Transfusion Evidence Library | From 1950  Clinical Speciality: Blood Donors and Donation Practice  Subject Area: Donor Care  Subject Area: Recruitment and Retention of Blood Donors |

**Appendix S3**. Study inclusion and exclusion criteria

| **Population** | **Include** studies of healthy voluntary whole blood donors ≥16 years of age |
| --- | --- |
|  | **Exclude** studies including any paid, replacement, apheresis, or autologous donors |
| **Exposure** | **Include** studies of observed risk factors for VVRs |
|  | **Exclude** studies of genetic risk factors for VVRs  **Exclude** studies of experimentally manipulated risk factors for VVRs |
| **Comparison** | N/A |
| **Outcome** | **Include** studies reporting risk factors for VVRs (as defined by ISBT guidelines)^1^ |
|  | **Exclude** studies reporting risk factors for any other donation complications |
| **Study design and type** | **Include** observational (cross-sectional, case-control, and cohort) and interventional (randomised controlled trial [RCT] and before-and-after) studies  **Include** English-language studies  **Include** studies conducted within any geographic setting |
|  | **Exclude** case reports, systematic, scoping, or rapid reviews, meta-analyses, conference abstracts, and abstract-only journal supplements |

**Appendix S4**. Modified Newcastle-Ottawa Scales for risk of bias assessment

| Cross-sectional studies | | | |
| --- | --- | --- | --- |
| Category | Criteria | Rating | Further information |
| Selection | 1. Is the outcome definition adequate? | a) Yes, with independent validation * | Independent validation may be conducted by study teams on-site to observe vasovagal reaction occurrences. |
|  |  | b) Yes, using record linkage * | Record linkage may be conducted using donation centre or blood service records. Importantly, records may include both donor- and phlebotomist-reported VVRs, but donor-reported VVRs will typically be confirmed by phlebotomists, who may also provide care to the donor. |
|  |  | c) Unsure, eg self report | Self-reporting may occur through questionnaires such as the BDRI or verbal interviews after donation and do not involve confirmation by phlebotomists or the blood service in general. |
|  |  | d) No description | Ascertainment of VVR occurrence was not described. |
|  | 1. Is the sample representative of the target population? | a) Yes, eg all subjects represented or random sampling * | Study participants may be truly representative of the average donor when studies include all donations (and therefore, all VVRs) that occur in a donation centre or blood service region during the investigation period.  True representativeness may also be achieved by the truly random selection of donations that occur in a donation centre or blood service region during the investigation period. |
|  |  | b) No, selected sample with potential for selection bias | Studies involving a relatively narrow group of donors such as those attending high school or university blood drives may be included in this category. Trials conducted in a non-representative group of donors may also be included in this category. |
|  |  | c) No description | Selection of the study sample was not described. |
| Comparability | 1. Comparability of different outcome groups on the basis of design or analysis | a) Study controls for no covariates | Key covariates of age, sex, and new (vs returning) donor status were selected on the basis of their consistent associations with VVR risk in the previous literature |
|  |  | b) Study controls for one of the following: age, sex, and new (vs returning) donor status * |  |
|  |  | c) Study controls for two or more of the following: age, sex, and new (vs returning) donor status ** |  |
| Exposure | 1. Non-response rate | a) None/same rate for different outcome groups * | Identical non-response rates across outcome groups are assumed when summary data indicate that no missing data is present. |
|  |  | b) Non-respondents described | No point is granted when the proportion of missingness in summary data is unclear or when a proportion of missingness is stated without description of differences or similarities across outcome groups. |
|  |  | c) No description |  |

| Case-control studies | | | |
| --- | --- | --- | --- |
| Category | Criteria | Rating | Further information |
| Selection^a^ | 1. Is the case definition adequate? | a) Yes, with independent validation * | Independent validation may be conducted by study teams on-site to observe vasovagal reaction occurrences. |
|  |  | b) Yes, using record linkage * | Record linkage may be conducted using donation centre or blood service records. Importantly, records may include both donor- and phlebotomist-reported VVRs, but donor-reported VVRs will typically be confirmed by phlebotomists, who may also provide care to the donor. |
|  |  | c) Unsure, eg self report | Self-reporting may occur through questionnaires such as the BDRI or verbal interviews after donation and do not involve confirmation by phlebotomists or the blood service in general. |
|  |  | d) No description | Ascertainment of VVR occurrence was not described. |
|  | 1. Representativeness of the cases | a) Consecutive or obviously representative series of cases * | A representative series of cases may be present when a study captures all donations (and therefore, VVRs) that occur in a donation centre or blood service region during the period of investigation using record linkage.  A representative series of cases may also occur when studies randomly select a series of VVR occurrences from all those that occur during the period of investigation. |
|  |  | b) Potential for selection biases or not stated | Selection biases may occur when studies select a series of VVR occurrences using convenience sampling or when studies select VVR cases with only complete information about risk factors. Possible biases may also arise in those studies that do not specify their case selection process. |
| Comparability | 1. Comparability of different outcome groups on the basis of design or analysis | a) Study controls for no covariates | Key covariates of age, sex, and new (vs returning) donor status were selected on the basis of their consistent associations with VVR risk in the previous literature. |
|  |  | b) Study controls for one of the following: age, sex, and new (vs returning) donor status * |  |
|  |  | c) Study controls for two or more of the following: age, sex, and new (vs returning) donor status ** |  |
| Exposure^b^ | 1. Non-response rate | a) Same rate for both groups * | Identical non-response rates for cases and controls are assumed when summary data indicate that no missing data is present. |
|  |  | b) Non respondents described | No point is granted when the proportion of missingness in summary data is unclear or when a proportion of missingness is stated without description of differences or similarities across case and control groups. |
|  |  | c) Rate different and no designation |  |

^a^Existing item of “selection of controls” was removed because all controls originated from community settings. Existing item of “definition of controls” was removed because disease (VVR) history was, in itself, a risk factor assessed in this synthesis. In addition, VVR history does not equate to disease history as defined in case-control studies of chronic disease because VVRs can only occur during a donation encounter and are not an underlying condition. Moreover, blood service guidelines differ with regards to their deferral of donors with previous VVRs^2-4^.

^b^Existing item of “ascertainment of exposure” was removed because meta-analysed risk factors were objectively measured (i.e., pre-donation blood pressure, heart rate, and haemoglobin) or were at little risk of recall bias due to outcome occurrence (i.e., sex, age, height, weight). Qualitatively synthesised risk factors were generally self-reported; quality assessment would not have differentiated between studies at high and low risk of bias. Existing item of “Same method of ascertainment for cases and controls” was removed because no variation was observed between studies; all studies used the same method of ascertainment for cases and controls.

| Cohort studies | | | |
| --- | --- | --- | --- |
| Category | Criteria | Rating | Further information |
| Selection^a^ | 1. Representativeness of the exposed cohort | a) Truly representative of the average donor in the community * | Study participants may be truly representative of the average donor when studies include all donations (and therefore, all VVRs) that occur in a donation centre or blood service region during the investigation period.  True representativeness may also be achieved by the truly random selection of donations that occur in a donation centre or blood service region during the investigation period. |
|  |  | b) Somewhat representative of the average donor in the community * | Studies involving donors from specific community groups such as the military may be included in this category. |
|  |  | c) Selected group of users eg nurses, volunteers | Studies involving a relatively narrow group of donors such as those attending high school or university blood drives may be included in this category. Trials conducted in a non-representative group of donors may also be included in this category. |
|  |  | d) No description of the derivation of the cohort | Methods of cohort selection were not described. |
|  | 1. Non-response rate | a) None/same rate for different cohorts * | Identical non-response rates for exposed and non-exposed cohorts were assumed when summary data indicate that no missing data is present. |
|  |  | b) Non-respondents described | No point was granted when the proportion of missingness in summary data was unclear or when a proportion of missingness is stated without description of differences or similarities across exposed and non-exposed cohorts. |
|  |  | c) No description | Non-response or missingness rates were not described. |
| Comparability | 1. Comparability of different outcome groups on the basis of design or analysis | a) Study controls for no covariates | Key covariates of age, sex, and new (vs returning) donor status were selected on the basis of their consistent associations with VVR risk in the previous literature |
|  |  | b) Study controls for one of the following: age, sex, and new (vs returning) donor status* |  |
|  |  | c) Study controls for two or more of the following: age, sex, and new (vs returning) donor status** |  |
| Outcome^b^ | 1. Assessment of outcome | a) Independent blind assessment * | Independent validation may be conducted by study teams on-site to observe vasovagal reaction occurrences. |
|  |  | b) Record linkage * | Record linkage may be conducted using donation centre or blood service records. Importantly, records may include both donor- and phlebotomist-reported VVRs, but donor-reported VVRs will typically be confirmed by phlebotomists, who may also provide care to the donor. |
|  |  | c) Self report | Self-reporting may occur through questionnaires such as the BDRI or verbal interviews after donation and do not involve confirmation by phlebotomists or the blood service in general. |
|  |  | d) No description | Ascertainment of VVR occurrence was not described. |

^a^Existing item of “selection of the non exposed cohort” was removed because no variation was observed between studies; all studies drew exposed and non-exposed cohorts from the same community. Existing item of “ascertainment of exposure” was removed because meta-analysed risk factors were objectively measured (i.e., predonation blood pressure, heart rate, and haemoglobin) or were at little risk of recall bias due to outcome occurrence (i.e., sex, age, height, weight). Qualitatively synthesised risk factors were generally self-reported; quality assessment would not have differentiated between studies at high and low risk of bias. Existing item of “demonstration that outcome of interest was not present at start of study” was removed because VVRs can only present during or after a donation (i.e., previous faints or pre-faints outside of the donation setting are irrelevant). By definition, a VVR cannot be present when an individual presents for donation (the “start of the study”). New item was added to NOS scale to assess biases associated with selective data missingness (similar to cross-sectional and case-control study NOS scales).

^b^Existing item of “was follow-up long enough for outcomes to occur” was removed because studies did not contain consistent descriptions of whether off-site VVRs were captured. Existing item of “adequacy of follow up of cohorts” was removed because all included studies had a specific aim of measuring VVR risk factors and therefore had largely complete follow-up for the outcome.

**Appendix S5**: Methods used to transform study-specific ratio estimates to common scale

Analyses of categorised continuous risk factors assumed that these factors were normally distributed and exhibited log-linear associations with VVRs.^5^ Summary data on donor weight and VVRs from Bravo and colleagues’ 2011 study^6^ is used to illustrate ratio estimate conversion methods.

| **Weight, kg** | **Midpoints (**$\boldsymbol{m}$**)** | **Whole blood donations (**$\boldsymbol{n}$**)** | **VVRs** |
| --- | --- | --- | --- |
| 49.9 – 54.0 | 51.95 | 16,639 | 46 |
| 54.4 – 58.5 | 56.45 | 34,791 | 79 |
| 59.0 – 65.3 | 62.15 | 76,466 | 142 |
| 65.8 – 70.0 | 67.9 | 55,904 | 59 |
| 70.3 – 90.3 | 80.3 | 218,765 | 184 |
| 90.7 – 117.5 | 104.1 | 128,907 | 72 |
| ≥117.9 | 131.3 | 22,802 | 11 |

Adapted from Table 1 of Bravo 2011.^6^ Midpoints for closed categories were calculated as averages of lower and upper bounds where means/medians of risk factor values were not available, while open-ended categories were calculated using half-widths of contiguous ranges.

1. We calculated the mean and standard deviation (SD) of the weight distribution in Bravo 2011 using the following formulae:

$$\hat{x}= \frac{\sum m*n}{\sum n}$$

$$s= \sqrt{\frac{\sum n*m^{2}}{\sum n}-\hat{x}^{2}}$$

2. We calculated ORs (approximating RRs) for risk factor values above and below any study-specified cutoff. Below is an example 2x2 table used to compare VVR odds between donors above and below 54 kg:

|  | VVR | No VVR |
| --- | --- | --- |
| >54 | 547 | 537,635 |
| ≤54 | 46 | 16,639 |

$$RR= OR=\frac{547\times16639}{46\times537635} =0.44$$

$$SElnRR= SElnOR= \frac{1}{547}+\frac{1}{537635}+ \frac{1}{46}+ \frac{1}{16639}=0.02$$

SElnOR: standard error of log odds ratio; SElnRR: standard error of log risk ratio

3. We converted cutoff comparison RRs and 95% CIs to top-vs-bottom-tertile RRs and 95% CIs (with risk factor ranges restricted to two SDs below the lowest mean risk factor value to two SDs above the highest mean risk factor value reported across studies) using previously described methods and software.^5^

| -1.09 | | | 0 | | | | 1.09 |
| --- | --- | --- | --- | --- | --- | --- | --- |
| -1.92 | | 0.14 | | | | | |
|  | |  | | |  | | |
|  | $c$  54 kg | | | $\hat{x}$  81.8 kg | |  | |

Assuming normally distributed weights and log-linear relationships between weight and VVRs, the log RR comparing the highest and lowest tertiles of weight values corresponded to 2.18 SDs higher weight, while the log RR comparing weight values above and below this study’s cutoff $c$ of 54 kg corresponded to 2.06 SDs higher weight.

We then used the below formulae to transform RRs and 95% CIs:

$$lnRR = ln(0.44)$$

$$cRR = e^{(lnRR*2.18/2.06)}$$

$$cLCI= e^{\left( \left( lnOR-1.96*SElnRR \right)*\frac{2.18}{2.06} \right)}$$

$$cUCI=e^{\left( \left( lnOR+1.96*SElnRR \right)*\frac{2.18}{2.06} \right)}$$

lnRR: log risk ratio; cRR: converted risk ratio; cLCI: converted lower 95% confidence interval of the risk ratio; cUCI: converted upper 95% confidence interval of the risk ratio

**Appendix S6**. Methods used to pool associations for age and VVRs

Case and total participant numbers across ≥3 age categories were required for dose-response analyses, and where unavailable, weighted least squares was used to calculate variances of per-unit risk factor estimates. Average exposures (“doses”) per risk factor category were calculated using range midpoints when per-category mean or median values were unavailable. For open-ended categories, half-widths of contiguous ranges^7^ or contextual donor eligibility criteria determined lower and upper bounds. In addition, non-log-linearity of age associations were explored through a restricted cubic spline (RCS)^8^ with three fixed knots at 10^th^, 50^th^, and 90^th^ percentiles of continuous age values.^9^

**Appendix S7**. Methods used to conduct sensitivity analyses for missing risk factor data

To evaluate the robustness of pooled unadjusted estimates to missing risk factor data in individual studies, summary-level data were imputed where available by assigning all VVR cases with missing risk factor values to exposed and unexposed categories (for binary risk factors) and lowest and highest exposure categories (for categorised continuous risk factors). These data were then used to generate the largest and smallest magnitude pooled estimates for each risk factor compatible with known missingness patterns using methods identical to main analyses, with these two estimates known subsequently as “best-case” and “worst-case” scenarios.

This approach is analogous to the best- and worst-case imputation strategy described by Higgins and colleagues in their general framework of strategies to handle missing outcomes in trial meta-analyses.^10^ Though these authors recommend resizing summary datasets following imputation to ensure that standard errors are not artificially reduced by the re-incorporation of missing observations,^10^ a simpler, unweighted method was used in this case for computational simplicity.

**Appendix S8**. Participant, outcome, and design characteristics of and risk factors examined by included studies

Table S1: Participant, outcome, and design characteristics of studies reporting meta-analysed risk factors

| Reference | Country(ies) | Study design | Number of donations | VVR incidence (%)^a^ | Donor age  (years; mean, SD/range)^a,b^ | Donor sex  (% female)^a^ | VVR assessment method^c^ | VVR definition |
| --- | --- | --- | --- | --- | --- | --- | --- | --- |
| Agarwal 2016^11^ | India | cohort | 30,928 | 2.2 | 28, 5.5 | 5.9 | P | syncope, presyncope |
| Almutairi 2017^12^ | Saudi Arabia | cohort | 18,936 | 1.1 | 26, 7.9 | 1 | P | syncope, presyncope |
| Bravo 2011^6^ | United States | cohort | 554,534 | 0.1 | >17 | 59 | P | syncope only |
| Broadwater 2021^13^ | United States | cohort | 1,514 | 2.8 | 27, NR | 68 | P | syncope, presyncope |
| Brunson 2022^14^ | United States | cohort | 130,889 | 4.4 | 37, 15.2 | 50 | P | syncope, presyncope |
| Burkhardt 2015^15^ | Germany | cohort | 1,004,942 | 0.07 | 44, NR | 48 | P | syncope, presyncope |
| Dunbar 2011^16^ | United States | cohort | 662 | 2.4 | NR, >17 | NR | P | syncope, presyncope |
| Eder 2008^17^ | United States | cohort | 1,776,445 | 3 | NR, >16 | 47 | P | syncope, presyncope |
| Fraile 2021^18^ | Spain | case-control | 315 | 0.15 | NR, 18-70 | 14 | P | syncope only |
| France 2005^19^ | United States | cohort | 89,544 | 8.5 | 38, 14.0 | 53 | P | syncope, presyncope |
| France 2009^20^ | United States | cohort | 65 | 35 | 22, NR | 52 | P | syncope, presyncope |
| France 2013^21^ | United States | cohort | 1,715 | 17.4 | NR, 17-18 | NR | P | syncope, presyncope |
| France 2019^22^ | United States | cohort | 2,716 | 12.1 | 17.3, 0.9 | 60 | P | syncope, presyncope |
| Gillet 2015^23^ | Belgium | cohort | 523,471 | 0.82 | NR, >18 | NR | P | syncope, presyncope |
| Goldman 2013^24^ | Canada | cohort | 18,108 | 7.4 | NR, >17 | 52 | P | syncope, presyncope |
| Goldman 2019^25^ | United States, United Kingdom, New Zealand, Australia, Canada | cohort | 10,214,048 | NR | NR, >24 | 45 (United States)  53 (United Kingdom)  54 (New Zealand)  36 (Australia)  44 (Canada) | P | syncope, presyncope |
| Goldman 2021^26^ | Canada | cohort | 772,505 | 0.2 | NR, >17 | 44 | P | syncope only |
| Hasan 2020^27^ | Malaysia | cohort | 27,890 | 1.5 | NR, 17-65 | 35 | P | syncope, presyncope |
| Hashizume 2023^28^ | Japan | cohort | 577,325 | 0.4 | NR, 17-69 | 29 | P | syncope, presyncope |
| Ibrahim 2023^29^ | Malaysia | case-control | 477 | 0.4 | NR, NR | 54 | P | syncope, presyncope |
| Inaba 2013^30^ | Japan | cross-sectional | 53,844 | 5.2 | 41.2, 12.2 | 27 | S | syncope, presyncope |
| Kuttath 2021^31^ | India | cohort | 953 | 10.2 | 27, 7 | 6 | P | syncope, presyncope |
| Majlessi 2008^32^ | Iran | cohort | 554 | 13.4 | 37, 10.6 | 6 | S | syncope, presyncope |
| Muller-Steinhardt 2012^33^ | Germany | cohort | 710,048 | 0.05 | NR, 18-70 | 41 | P | syncope, presyncope |
| Newman 2002^34^ | United States | cohort | 1,076 | 8.2 | NR, >16 | 49 | P | syncope, presyncope |
| Newman 2003^35^ | Canada | cohort | 2,022 | 5.5 | 17, NR | 52 | P | syncope, presyncope |
| Newman 2006^36^ | United States | cohort | 7,274 | 12 | 17, NR | 50 | P | syncope, presyncope |
| Newman 2007^37^ | United States | cohort | 8,894 | 11.4 | NR, >16 | 48 | P | syncope, presyncope |
| Nilsson Sojka 2003^38^ | Sweden | cross-sectional | 528 | 7.2 | 39, NR | 40 | S | syncope, presyncope |
| Philip 2014^39^ | India | cohort | 88,201 | 1.2 | NR, NR | 4 | P | syncope, presyncope |
| Pisciotto 1982^40^ | United States | cohort | 16,424 | 3.3 | NR, NR | NR | P | syncope, presyncope |
| Reiss 2009^41^ | United States | cohort | 72,769 | 8.2 | NR, 16-29 | 50 | P | syncope, presyncope |
| Rios 2010^42^ | United States | cohort | 591,177 | 3.8 | NR, 16-70 | 54 | P | syncope, presyncope |
| Sachdev 2017^43^ | India | cohort | 1,000 | 2.5 | 20, 2.6 | 15 | P | syncope, presyncope |
| Schmidt 1975^44^ | United States | cross-sectional | 42 | 9 | NR, NR | 55 | P | syncope, presyncope |
| Takanashi 2012^45^ | Japan | case-control | 27,270 (400mL donations only) | 0.4 | 35, 11.8 | 24 | P | syncope, presyncope |
| Thijsen 2020^46^ | Australia | cohort | 382,628 | 2.1 | 44, 15.9 | 51 | P | syncope, presyncope |
| Tomasulo 2011^47^ | United States | cohort | 112,743 (2008 donations only) | 2.5 | NR, 17-22 | 56 | P | syncope, presyncope |
| van den Berg 2012^48^ | South Africa | cohort | 2,466 | 1.7 | NR, 16-20 | 38 | P | syncope, presyncope |
| van den Hurk 2017^49^ | The Netherlands | cohort | 23,064 | 4.6 | NR, 18-79 | 52 | S | syncope, presyncope |
| Vavic 2014^50^ | Serbia | cohort | 3,646 | 3.2 | NR, 18-19 | 37 | P | syncope, presyncope |
| Veldhuizen 2012^51^ | The Netherlands | cross-sectional | 12,051 | 2.6 | 45, NR | 53 | S | syncope, presyncope |
| Wang 2019^52^ | Taiwan SAR | case-control | 6,338 | 0.1 | NR, 20-65 | 41 | P | syncope, presyncope |
| Wiersum-Osselton 2014^53^ | The Netherlands | cohort | 551,744 | 0.8 | 46, NR | 42 | P | syncope, presyncope |
| Wiersum-Osselton 2019^54^ | The Netherlands | cohort | 8,300 | 18.5 | NR, <30 | 70 | S | syncope, presyncope |
| Wiltbank 2008^55^ | United States | cohort | 422,231 | 1.4 | NR, >17 | 58 | P | syncope, presyncope |
| Wong 2013^56^ | Hong Kong SAR | cohort | 38,436 | 4.4 | NR, 16-18 | 43 | P | syncope, presyncope |

^a^NR: not reported. ^b^SD: standard deviation. ^c^P: phlebotomist-reported; S: self-reported.

Goldman 2019 was treated as five separate studies for the purposes of meta-analyses.

Table S2: Risk factors synthesised in meta-analysed studies

| Reference | Meta-analysed risk factors |
| --- | --- |
| Agarwal 2016^11^ | Sex. |
| Almutairi 2017^12^ | Age: <30 y, ≥30 y; DBP: ≤89 mmHg, >89 mmHg; haemoglobin: 12.5-13 g/dL, 13.1-14 g/dL, 14.1-15 g/dL, >15 g/dL; new (vs returning) donor status; SBP: ≤139 mmHg, >139 mmHg; sex. |
| Bravo 2011^6^ | Age: 17-18 y, 19-22 y, 23-24 y, 25-65 y, >65 y; BMI: <18.5 kg/m^2^, 18.5-22.49 kg/m^2^, 22.50-24.99 kg/m^2^, 25.00-29.99 kg/m^2^, 30-39 kg/m^2^; DBP: <70 mmHg, 70-85 mmHg, >85 mmHg; donation site, EBV: <3.5 L, 3.5-3.999 L, 4-4.499 L, 4.5-4.999 L, >5 L; height: <1.47 m, 1.47-1.52 m, 1.55-1.63 m, 1.65-1.7 m, 1.73-1.83 m, >1.83 m; heart rate: <65 bpm, 65-90 bpm, >90 bpm; new (vs returning) donor status; race/ethnicity, SBP: <100 mmHg, 100-140 mmHg, >140 mmHg; sex; weight: 49.9-54 kg, 54.4-58.5 kg, 59-65.3 kg, 65.8-70 kg, 70.3-90.3 kg, 90.7-117.5 kg, >117.9 kg. |
| Broadwater 2021^13^ | Heart rate: <50 bpm, 50-100 bpm. |
| Brunson 2022^14^ | Age: <20 y, 20-29 y, 30-39 y, 40-49 y, 50-59 y, 60-69 y, >70 y; haemoglobin: 12-13.5 g/dL, 13.5-14.4 g/dL, 14.4-15.4 g/dL, ≥15.4 g/dL; sex. |
| Burkhardt 2015^15^ | New (vs returning) donor status |
| Dunbar 2011^16^ | Age: ≤22 y, >22 y. |
| Eder 2008^17^ | Age: 16-17 y, 18-19 y, ≥20 y; new (vs returning) donor status; sex. |
| Fraile 2021^18^ | Heart rate: ≤60 bpm, >60 bpm; new (vs returning) donor status; SBP: ≤120 mmHg, >120 mmHg; weight: ≤65 kg, >65 kg. |
| France 2005^19^ | New (vs returning) donor status; race/ethnicity; sex. |
| France 2009^20^ | Sex. |
| France 2013^21^ | Sex. |
| France 2019^22^ | Sex. |
| Gillet 2015^23^ | New (vs returning) donor status; sex. |
| Goldman 2013^24^ | Age: 17-22 y, 23-29 y, 30-49 y, ≥50 y; new (vs returning) donor status; sex. |
| Goldman 2019^25^ | Age: 24-70 y, ≥71 y. |
| Goldman 2021^26^ | New (vs returning) donor status; sex. |
| Hasan 2020^27^ | Age: 17-25 y, 26-35 y, 36-45 y, 46-65 y; new (vs returning) donor status; donation site; sex. |
| Hashizume 2023^28^ | Age: 17-19 y, 20-29 y, 30-49 y, 50-69 y; DBP: ≤59 kg, 60-79 kg, 80-99 kg, ≥100 kg; donation site; EBV: ≤3.499 L, 3.5-4.499 L, 4.5-5.499 L, 5.5 L; height: ≤1.59 m, 1.60-1.79 m, ≥1.8 m; heart rate: ≤59 bpm, 60-89 bpm, ≥90 bpm; new (vs returning) donor status; SBP: 90-99 mmHg, 100-119 mmHg, 120-139 mmHg, 140-159 mmHg, ≥160 mmHg; sex; weight: 50-59 kg, 60-69 kg, 70-79 kg, 80-89 kg, ≥90 kg. |
| Ibrahim 2023^29^ | Age: <30 y, ≥30 y; donation site; new (vs returning) donor status; sex; weight: <55 kg, ≥55 kg. |
| Inaba 2013^30^ | Age: 18-19 y, 20-29 y, 30-39 y, 40-49 y, 50-59 y, 60-69 y; EBV: <4 L, 4-4.499 L, ≥4.5L; haemoglobin: <13.5 g/dL, ≥13.5 g/dL; new (vs returning) donor status; sex. |
| Kuttath 2021^31^ | Age: ≤19 y, 20-29 y, 30-39 y, ≥40 y; BMI: 18.5-25 kg/m^2^, 25-30 kg/m^2^, 30-35 kg/m^2^; sex. |
| Majlessi 2008^32^ | New (vs returning) donor status. |
| Muller-Steinhardt 2012^33^ | Age: 18-29 y, 30-39 y, 40-49 y, 50-59 y, 60-65 y, 66-68 y, 69-70 y; sex. |
| Newman 2002^34^ | New (vs returning) donor status; sex; weight: 49.9-59 kg, 59.4-68 kg, 68.5-81.2 kg, >81.6 kg. |
| Newman 2003^35^ | New (vs returning) donor status; weight: 49.9-63 kg, 63.5-76.6 kg, 77.1-90.3 kg, >90.7 kg. |
| Newman 2006^36^ | Sex; weight: 49.9-58.5 kg, 59-67.6 kg, 68-76.6 kg, 77.1-85.7 kg, 86.2-94.8 kg, ≥95.2 kg. |
| Newman 2007^37^ | New (vs returning) donor status; race/ethnicity; sex; weight: 49.9-58.5 kg, 59-67.6 kg, 68-76.6 kg, 77.1-85.7 kg, 86.2-94.8 kg, 95.2-103.9 kg, >104.3 kg. |
| Nilsson Sojka 2003^38^ | Age: <25 y, 25-34 y, 35-44 y, 45-54 y, 55-65 y; sex. |
| Philip 2014^39^ | Age: <45 y, ≥45 y; weight: <55 kg, ≥55 kg. |
| Pisciotto 1982^40^ | New (vs returning) donor status. |
| Reiss 2009^41^ | Age: 16 y, 17 y, 18 y, 19 y, 20-29 y; sex. |
| Rios 2010^42^ | Age: 16 y, 17 y, 18 y, 19-22 y, 23-69 y, 70 y; EBV: <3.5 L, 3.5-3.999 L, 4-4.499 L, 4.5-5 L, ≥5L; height: <1.52 m, 1.52-1.55 m, 1.57-1.60 m, 1.62-1.65 m, 1.68 m; new (vs returning) donor status; race; sex; weight: 49.9-54 kg, 54.4-58.5 kg, 59-63 kg, 63.5-67.6 kg, 68-78.9 kg, 79.4-90.3 kg, >90.7 kg. |
| Sachdev 2017^43^ | Age: 18-20y, 21-23y, >23y; BMI: <22 kg/m^2^, 22-25 kg/m^2^, >25 kg/m^2^; weight: 45-54 kg, 55-64 kg, 65-74 kg, >75 kg. |
| Schmidt 1975^44^ | Sex. |
| Takanashi 2012^45^ | Age: 18-19 y, 20-29 y, 30-39 y, 40-49 y, ≥50 y; BMI: <25 kg/m^2^, ≥25 kg/m^2^; DBP: <70 mmHg, ≥70 mmHg; EBV: <4.3 L, ≥4.3 L; heart rate: <90 bpm, ≥90 bpm; SBP: <100 mmHg, ≥100 mmHg; new (vs returning) donor status; sex. |
| Thijsen 2020^46^ | New (vs returning) donor status. |
| Tomasulo 2011^47^ | Sex. |
| van den Berg 2012^48^ | Age: ≤16 y, 17 y, 18-20 y; new (vs returning) donor status; race/ethnicity; sex. |
| van den Hurk 2017^49^ | Sex. |
| Vavic 2014^50^ | DBP: 60-64 mmHg, 65-75 mmHg, >80 mmHg; SBP: 100-105 mmHg, 105-135 mmHg, >140 mmHg; sex. |
| Veldhuizen 2012^51^ | Sex. |
| Wang 2019^52^ | Age: <35 y, ≥35 y; BMI: <24 kg/m^2^, ≥24 kg/m^2^; DBP: <75 mmHg, ≥75 mmHg; donation site; EBV: <4.085 L, ≥4.085 L; new (vs returning) donor status; SBP: <124 mmHg, ≥124 mmHg; sex. |
| Wiersum-Osselton 2014^53^ | Age: 18-19 y, 20-24 y, 25-34 y, 35-69 y; DBP: <60 mmHg, 60-90 mmHg, >90 mmHg; haemoglobin: 12.6-13.4 g/dL, 13.5015 g/dL, >15 g/dL (females), 13.5-14.3 g/dL, 14.5-16 g/dL, >16 g/dL (males); new (vs returning) donor status; SBP: <100 mmHg, 100-160 mmHg, >160 mmHg; sex: weight: <70 kg, ≥70 kg. |
| Wiersum-Osselton 2019^54^ | Sex. |
| Wiltbank 2008^55^ | Age: 17-18 y, 19-24 y, 25-65 y, >65 y; BMI: <18.5 kg/m^2^, 18.5-22.49 kg/m^2^, 22.5-24.99 kg/m^2^, 25-29.9 kg/m^2^, >30 kg/m^2^; DBP: <70 mmHg, 70-85 mmHg, >85 mmHg; EBV: <3.5 L, 3.5-4 L, 4.001-4.775 L, >4.775 L; heart rate: <65 bpm, 65-90 bpm, >90 bpm; race/ethnicity; SBP: <100 bpm, 100-140 bpm, >140 bpm. |
| Wong 2013^56^ | Sex; weight: 41-49 kg, 50-54 kg, 55-59 kg, 60-64 kg, ≥65 kg. |

Table S3: Participant, outcome, and design characteristics of studies included only in narrative syntheses

| Reference | Country(ies) | Study design | Number of donations | VVR incidence (%)^a^ | Donor age  (years; mean, SD or range)^a,b^ | Donor sex  (% female)^a^ | VVR assessment method^c^ | VVR definition |
| --- | --- | --- | --- | --- | --- | --- | --- | --- |
| Ditto 1995^57^ | Canada, United States | cohort | 483 | NR | 23, 0.3 | 62 | P | syncope, presyncope |
| Ditto 2006^58^ | Canada | cohort | 671 | NR | 20, 3.9 | 63 | P | syncope, presyncope |
| Ditto 2012a^59^ | Canada | cross-sectional | 1,002 | NR | 21, 3.9 | 60 | P | syncope, presyncope |
| Ditto 2012b^60^ | Canada | cohort | 88 | 11 | 20.4, 0.2 | 50 | P | syncope, presyncope |
| Ditto 2014^61^ | Canada | cohort | 1,202 | 13.6 | 22, 3.4 | 50 | P | syncope, presyncope |
| Eder 2012^62^ | United States | cohort | 378,692 | 6 | NR, >16 | NR | P | syncope, presyncope |
| Ferguson 2001^63^ | United Kingdom | cross-sectional | 349 | NR | 33, 13.1 | 52 | S | syncope, presyncope |
| France 2012^64^ | United States | cohort | 982 | NR | 45, 16.3 | 46 | S | syncope, presyncope |
| France 2016^65^ | United States | cohort | 2,730 | 11.7 | NR, 17-18 | 55 | P | syncope, presyncope |
| France 2020^66^ | United States | cohort | 4,035 | 6.4 | 17, 0.8 | 49 | P | syncope, presyncope |
| France 2021^67^ | United States | cohort | 872 | 13.6 | NR, 16-19 | 61 | P | syncope, presyncope |
| Kaloupek 1985^68^ | Canada | case-control | 48 | NR | 22, NR | 37 | P | syncope, presyncope |
| Krumholz 1997^69^ | United States | cohort | 329,143 | 2.24 | 34, NR | 52 | P | syncope, presyncope |
| Labus 2000^70^ | United States | cohort | 362 | NR | 22, 7.1 | 58 | S | syncope, presyncope |
| Meade 1996^71^ | United States | cohort | 311 | NR | 20, 2.9 | 64 | S | syncope, presyncope |
| Mennitto 2019^72^ | Canada | cross-sectional | 547 | NR | 21, 0.2 | 52 | P | syncope, presyncope |
| Mennitto 2020^73^ | Canada | cohort | 160 | 14.8 | 21, 0.2 | 54 | P | syncope, presyncope |
| Mennitto 2021^74^ | Canada | cross-sectional | 530 | 11.8 | 21, 0.1 | 51 | S | syncope, presyncope |
| Rudokaite 2023a^75^ | The Netherlands | case-control | 227 | NR | NR, NR | NR | S | syncope, presyncope |
| Rudokaite 2023b^76^ | The Netherlands | case-control | 193 | NR | NR, NR | 59 | S | syncope, presyncope |
| Stewart 2006^77^ | United States | Cohort | 8,869 | 5.4 | 41, NR | 49 | P | syncope, presyncope |
| Thijsen 2023^78^ | Australia | cohort | 442 | 12.7 | 30.7, 11.7 | 51 | P | syncope, presyncope |
| Viar 2010^79^ | United States | cohort | 446 | NR | 35.7, 14.1 | 64 | S | syncope, presyncope |
| Vossbeck-Elsebusch 2012^80^ | Germany | cross-sectional | 347 | NR | 24.4, 4.2 | 64 | S | syncope, presyncope |

^a^NR: not reported. ^b^SD: standard deviation. ^c^P: phlebotomist-reported; S: self-reported.

Studies excluded from quantitative syntheses due to lack of consistent exposure definitions (i.e., differing questionnaires to assess similar constructs), lack of consistent effect size measures (i.e., correlation coefficients rather than ratio measures), or lack of consistent outcome definitions (i.e., one-point increases in BDRI score instead of binary variable for VVR occurrence).

**Appendix S9**: Modified NOS scores of included studies

Table S4: Modified NOS scores of studies reporting meta-analysed risk factors

| Cross-sectional studies |  |  |  |  |
| --- | --- | --- | --- | --- |
| Reference | Selection | Comparability^a^ | Exposure | Total score |
| Inaba 2013 | 0 | 2 | 0 | 2 |
| Nilsson Sojka 2003 | 0 | 0 | 0 | 0 |
| Schmidt 1975 | 2 | 0 | 1 | 3 |
| Veldhuizen 2012 | 0 | 0 | 0 | 0 |
| Case-control studies | | | | |
| Reference | Selection | Comparability^a^ | Exposure | Total score |
| Fraile 2021 | 2 | 2 | 1 | 5 |
| Ibrahim 2023 | 2 | 2 | 0 | 4 |
| Takanashi 2012 | 1 | 1 | 0 | 2 |
| Wang 2019 | 2 | 2 | 0 | 4 |
| Cohort studies | | | | |
| Reference | Selection | Comparability^a^ | Outcome | Total score |
| Agarwal 2016 | 2 | 0 | 1 | 3 |
| Almutairi 2017 | 2 | 2 | 1 | 5 |
| Bravo 2011 | 1 | 2 | 1 | 4 |
| Broadwater 2021 | 2 | 0 | 1 | 3 |
| Brunson 2022 | 1 | 2 | 1 | 4 |
| Burkhardt 2015 | 2 | 0 | 1 | 3 |
| Dunbar 2011 | 2 | 0 | 1 | 3 |
| Eder 2008 | 2 | 0 | 1 | 3 |
| France 2005 | 1 | 0 | 1 | 2 |
| France 2009 | 1 | 0 | 1 | 2 |
| France 2013 | 1 | 0 | 1 | 2 |
| France 2019 | 0 | 0 | 1 | 1 |
| Gillet 2015 | 1 | 0 | 1 | 2 |
| Goldman 2013 | 0 | 0 | 0 | 0 |
| Goldman 2019 | 2 | 0 | 1 | 3 |
| Goldman 2021 | 2 | 0 | 1 | 3 |
| Hasan 2020 | 2 | 0 | 1 | 3 |
| Hashizume 2023 | 1 | 0 | 1 | 2 |
| Kuttath 2021 | 0 | 0 | 1 | 1 |
| Majlessi 2008 | 1 | 2 | 0 | 3 |
| Muller-Steinhardt 2012 | 2 | 0 | 1 | 3 |
| Newman 2002 | 1 | 0 | 1 | 2 |
| Newman 2003 | 2 | 0 | 1 | 3 |
| Newman 2006 | 2 | 0 | 1 | 3 |
| Newman 2007 | 1 | 0 | 1 | 2 |
| Philip 2014 | 2 | 2 | 1 | 5 |
| Pisciotto 1982 | 1 | 0 | 1 | 2 |
| Reiss 2009 | 2 | 0 | 1 | 3 |
| Rios 2010 | 1 | 2 | 1 | 4 |
| Sachdev 2017 | 1 | 0 | 1 | 2 |
| Thijsen 2020 | 2 | 0 | 1 | 3 |
| Tomasulo 2011 | 1 | 2 | 1 | 4 |
| van den Berg 2012 | 1 | 2 | 1 | 4 |
| van den Hurk 2017 | 0 | 2 | 0 | 2 |
| Vavic 2014 | 1 | 0 | 1 | 2 |
| Wiersum-Osselton 2014 | 1 | 1 | 1 | 3 |
| Wiersum-Osselton 2019 | 0 | 0 | 0 | 0 |
| Wiltbank 2008 | 1 | 2 | 1 | 4 |
| Wong 2013 | 2 | 0 | 1 | 3 |

^a^Though NOS scores for “comparability” (i.e., covariate adjustment) are displayed here for the reader’s information, these scores were not used in subgroup analyses comparing unadjusted estimates from studies with greater than vs less than median NOS scores.

Table S5: Modified NOS scores of studies reporting exclusively narratively synthesised risk factors

| Cross-sectional studies |  |  |  |  |
| --- | --- | --- | --- | --- |
| Reference | Selection | Comparability | Exposure | Total score |
| Ditto 2012 | 1 | 2 | 0 | 3 |
| Ferguson 2001 | 0 | 1 | 1 | 2 |
| France 2012 | 0 | 2 | 1 | 3 |
| Labus 2000 | 0 | 0 | 1 | 1 |
| Mennitto 2019 | 2 | 2 | 1 | 5 |
| Mennitto 2021 | 0 | 1 | 1 | 2 |
| Vossbeck-Elsebusch 2012 | 0 | 1 | 0 | 1 |
| Case-control studies | | | | |
| Reference | Selection | Comparability | Exposure | Total score |
| Kaloupek 1985 | 1 | 1 | 1 | 3 |
| Rudokaite 2023a | 0 | 0 | 1 | 1 |
| Rudokaite 2023b | 0 | 0 | 1 | 1 |
| Cohort studies | | | | |
| Reference | Selection | Comparability | Outcome | Total score |
| Ditto 1995 | 1 | 1 | 1 | 3 |
| Ditto 2012 | 1 | 1 | 1 | 3 |
| Ditto 2006 | 0 | 0 | 1 | 1 |
| Ditto 2014 | 0 | 0 | 1 | 1 |
| Eder 2012 | 2 | 0 | 1 | 3 |
| France 2016 | 1 | 0 | 1 | 2 |
| France 2020 | 1 | 0 | 1 | 2 |
| France 2021 | 0 | 0 | 1 | 1 |
| Krumholz 1997 | 2 | 0 | 1 | 3 |
| Meade 1996 | 0 | 0 | 0 | 0 |
| Mennitto 2020 | 1 | 0 | 1 | 2 |
| Stewart 2006 | 0 | 1 | 1 | 2 |
| Thijsen 2023 | 1 | 2 | 1 | 4 |
| Viar 2010 | 1 | 0 | 0 | 1 |

**Appendix S10**: Pooled covariate-adjusted risk factor associations

Figure S1: Covariate-adjusted associations between VVRs and (1) sex, (2) EBV, and (3) new (vs returning) donor status

(1) Sex


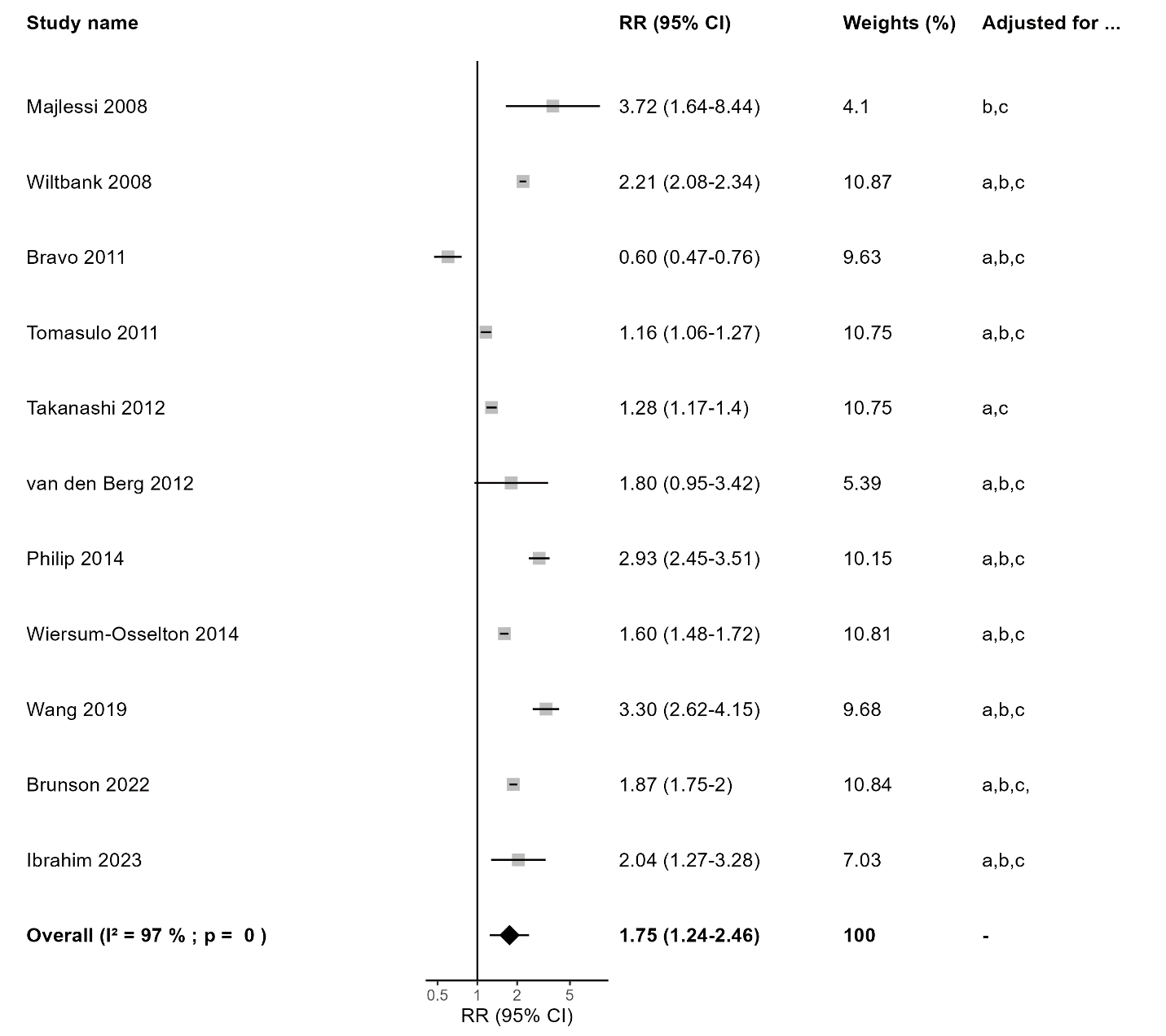


^a^Age; ^b^new (vs returning) donor status; ^c^other covariates.

(2) EBV


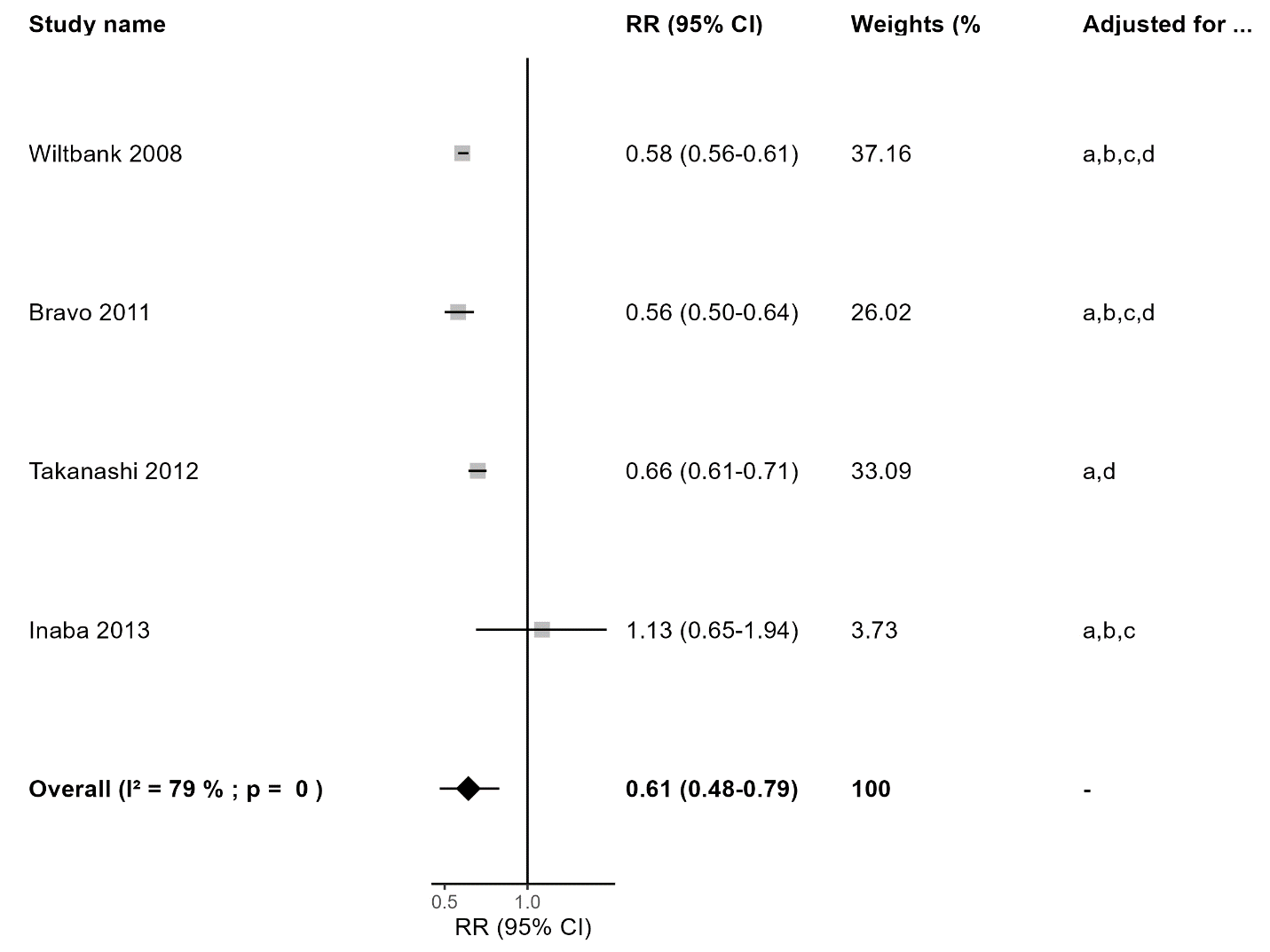


^a^Age; ^b^sex; ^c^new (vs returning) donor status; ^d^other covariates.

(3) New (vs returning) donor status


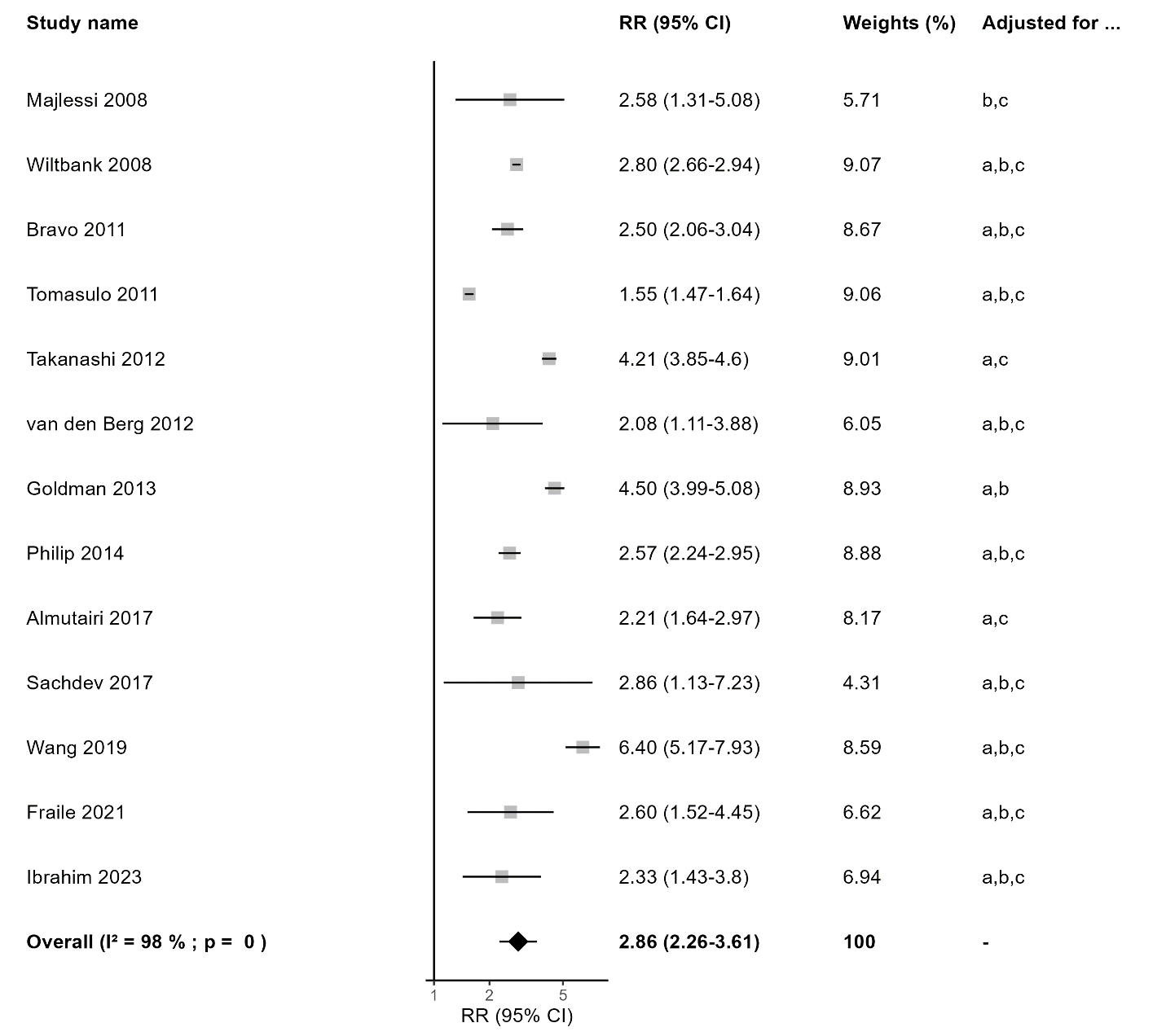


^a^Age; ^b^sex; ^c^other covariates.

**Appendix S11**. Findings from narrative syntheses

| **Risk factor category** | **Risk factor** | **Findings** |
| --- | --- | --- |
| Donation-specific factors | Number of previous donations | Positive association^30,38^ |
|  | History of donation-related VVRs | Positive association^23,28,62^ |
|  | Observing a VVR during donation | Positive association^61,74^ |
|  | Attended by phlebotomist with poorer interpersonal skills | Positive association^77^ |
|  | Longer blood draw time | Positive association^32,66^ |
|  | Greater changes in respiration rate and smaller changes in DBP during donation | Positive association^73^ |
|  | Higher-intensity pre-donation eye movements | Positive association^75^ |
|  | Higher-intensity pre-donation facial temperature fluctuations | Positive association^76^ |
| Psychologic factors | Fear of blood, injury, mutilation, or injection | Positive association ^21,22,30,59,64-67,70-72,79,80^ |
|  | Donation anxiety | Positive association^30,58,71,79^  Null association^44,68^ |
|  | Anxiety sensitivity | Positive association^71^ |
|  | Disgust sensitivity | Positive association^79,80^ |
|  | Perceived blood loss | Positive association^60^ |
|  | Neuroticism | Positive association^44,63^ |
|  | “Scared, fearful, and afraid” emotions | Positive association^78^ |
| Donor medical history | Hypertension/prehypertension | Negative association^30^  Null association^12^ |
|  | Family history of hypertension | Negative association^57^ |
|  | Non-beta-blocker antihypertensive use | Null association^40^ |
|  | Epilepsy diagnosis | Null association^69^ |
|  | Fainting history | Positive association^30,44,68^ |
|  | Acute or chronic health condition | Positive association^68^ |
| Miscellaneous factors | Short (<6 hours) sleep | Positive association^45^ |
|  | Short (≤5 hours) and long (≥9 hours) sleep | Positive association^28^ |
|  | Hunger | Positive association^30,45^ |
|  | Thirst | Positive association^30,45^ |
|  | Caffeine intake | Negative association^71^ |
|  | Number of hours since last meal | Null association^68^ |
|  | Amount of food consumed during last meal | Null association^68^ |
|  | Hot (vs non-hot) weather season | Null association^52^ (study in sub-tropical climate) |

**Appendix S12**. Subgroup analyses

Figure S2: Meta-analyses of unadjusted sex estimates across subgroups of ≥3 studies or participant subsets


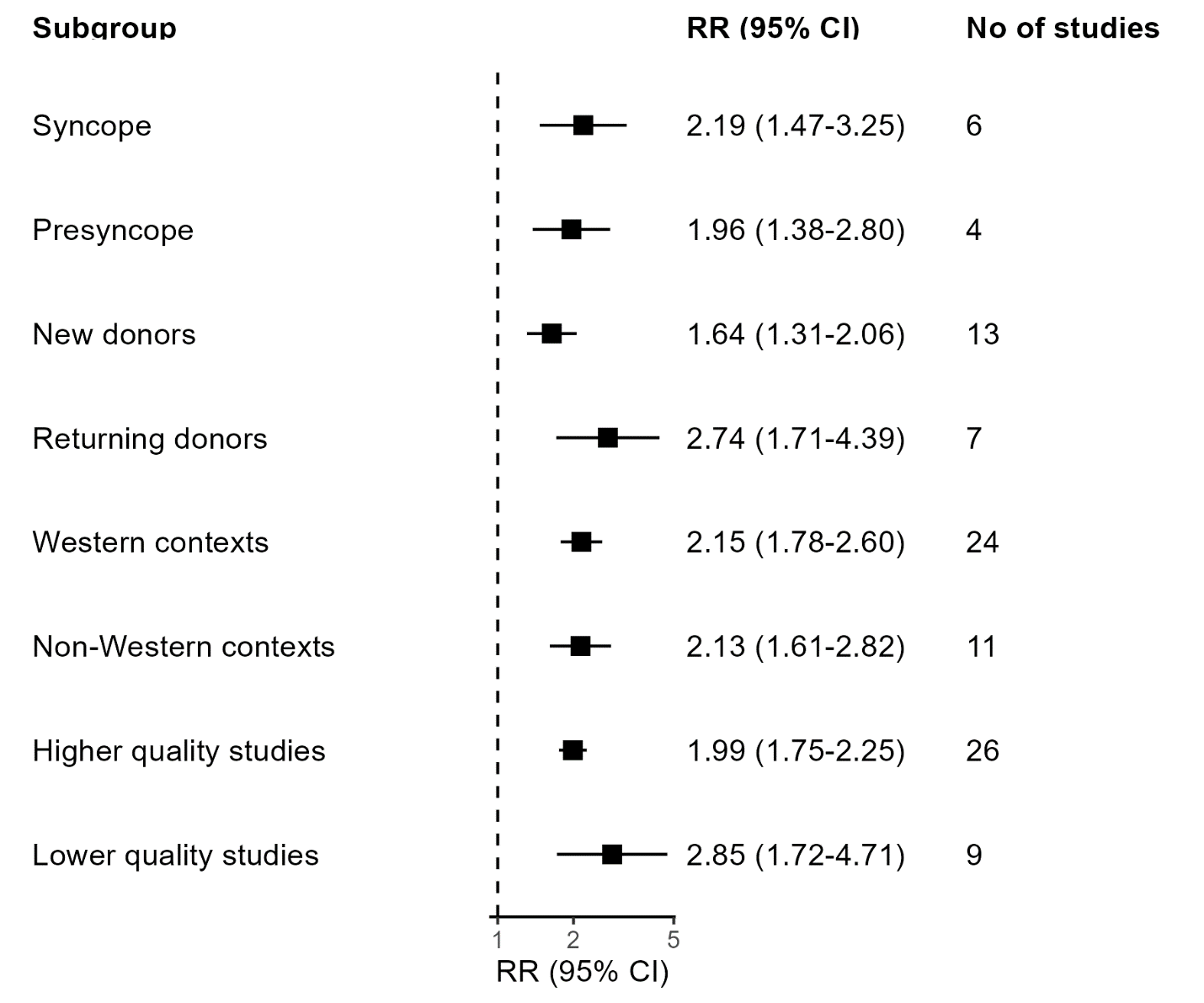


Figure S3: Meta-analyses of unadjusted age estimates across subgroups of ≥3 studies or participant subsets


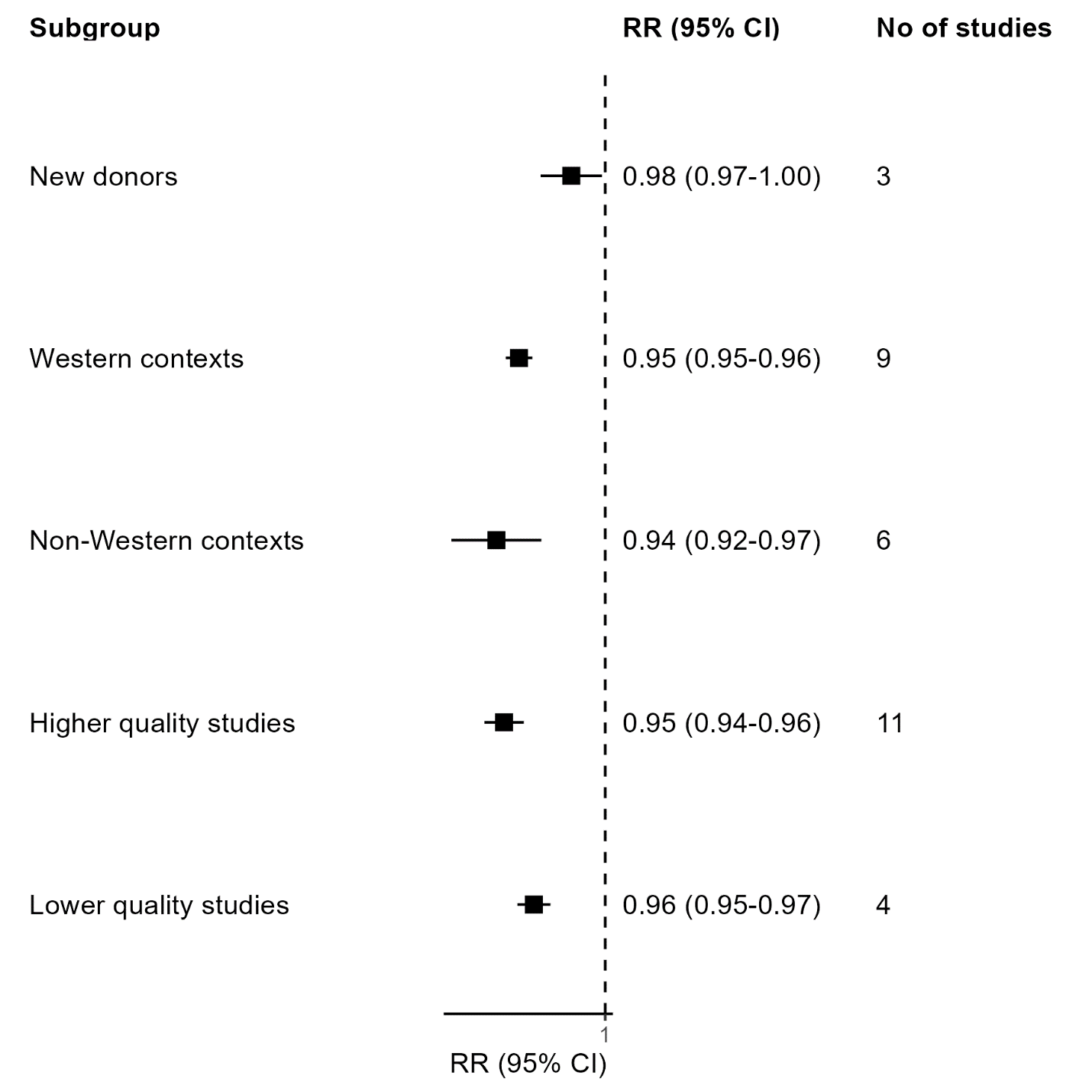


Figure S4: Meta-analyses of unadjusted weight estimates across subgroups of ≥3 studies or participant subsets


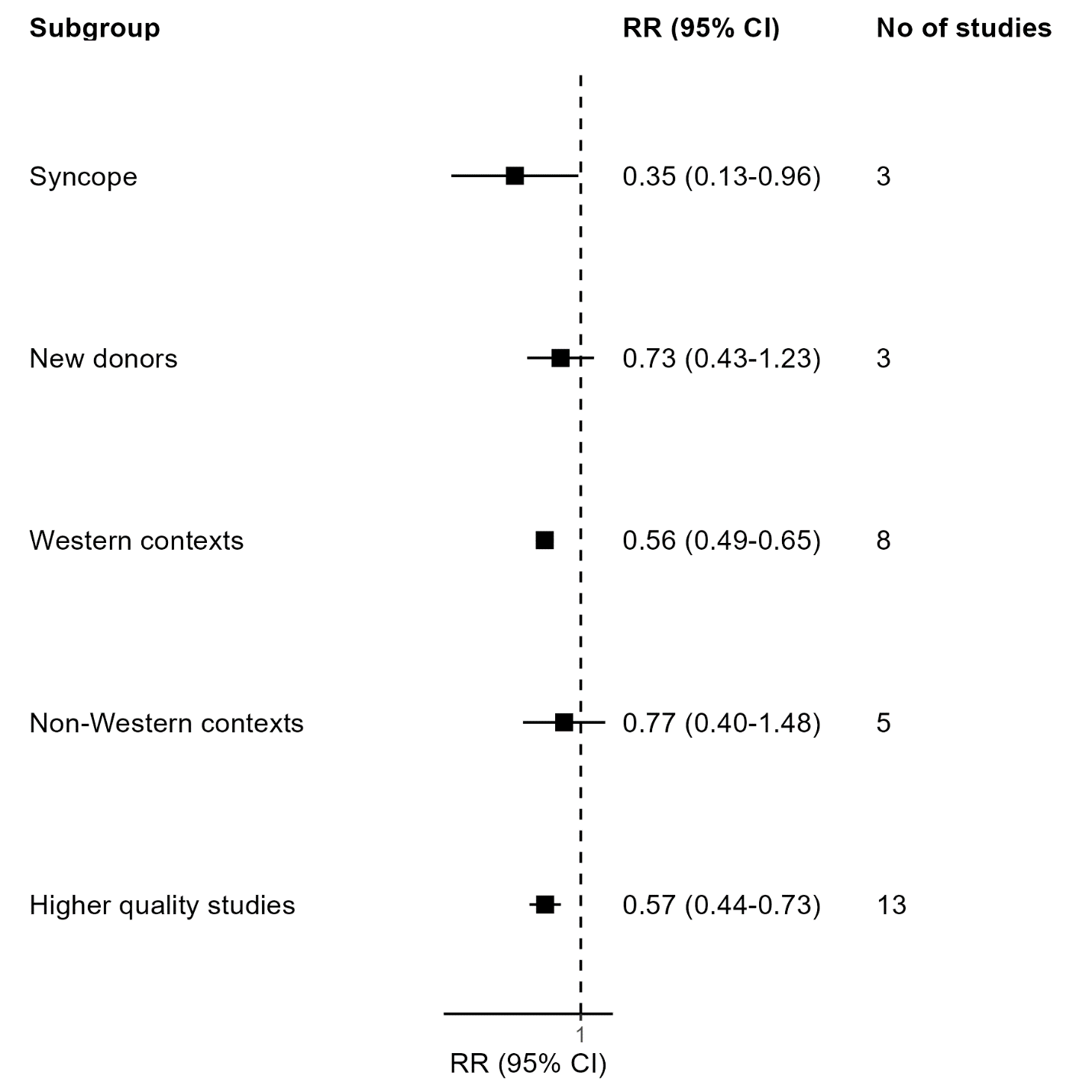


Figure S5: Meta-analyses of unadjusted EBV estimates across subgroups of ≥3 studies or participant subsets


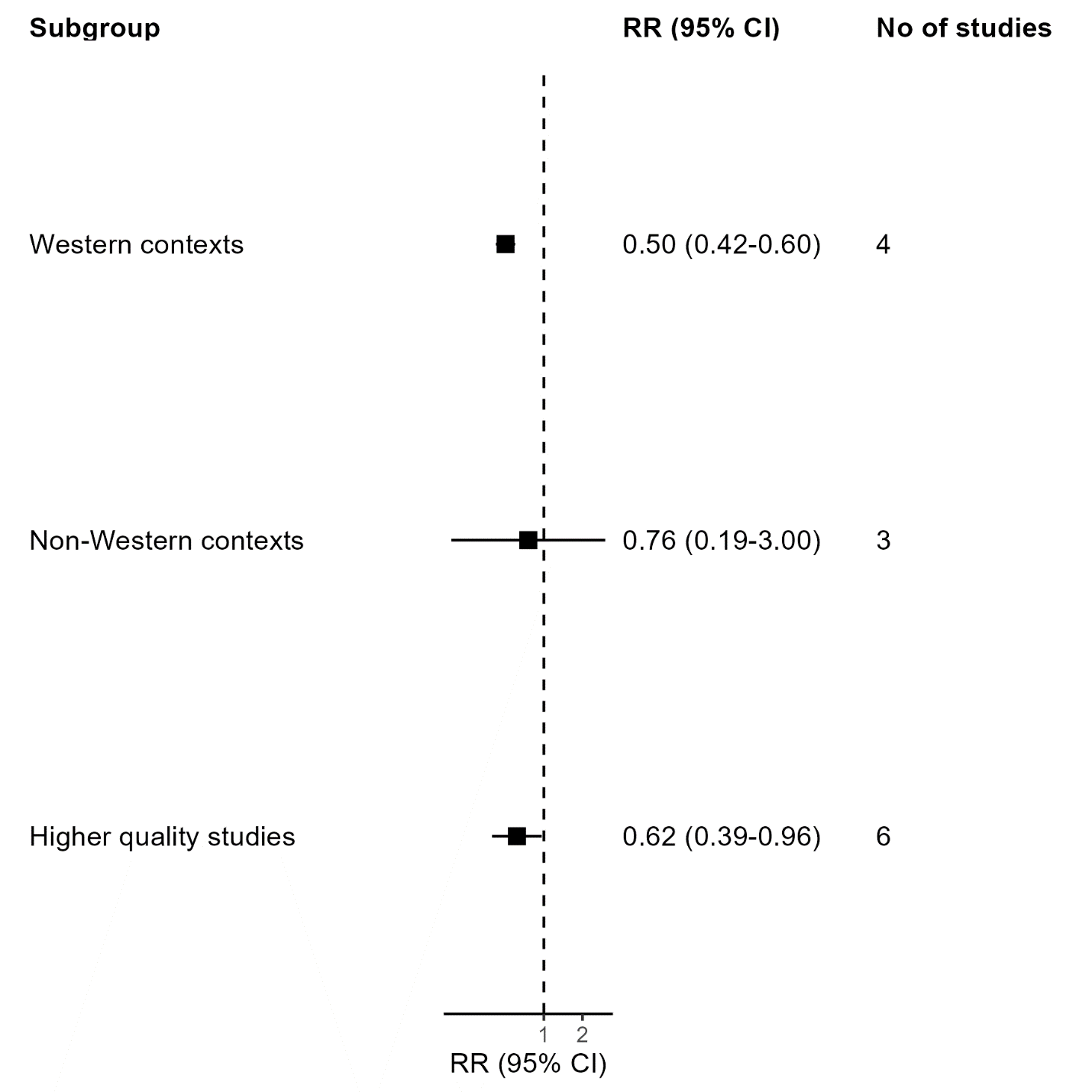


Figure S6: Meta-analyses of unadjusted SBP estimates across subgroups of ≥3 studies or participant subsets


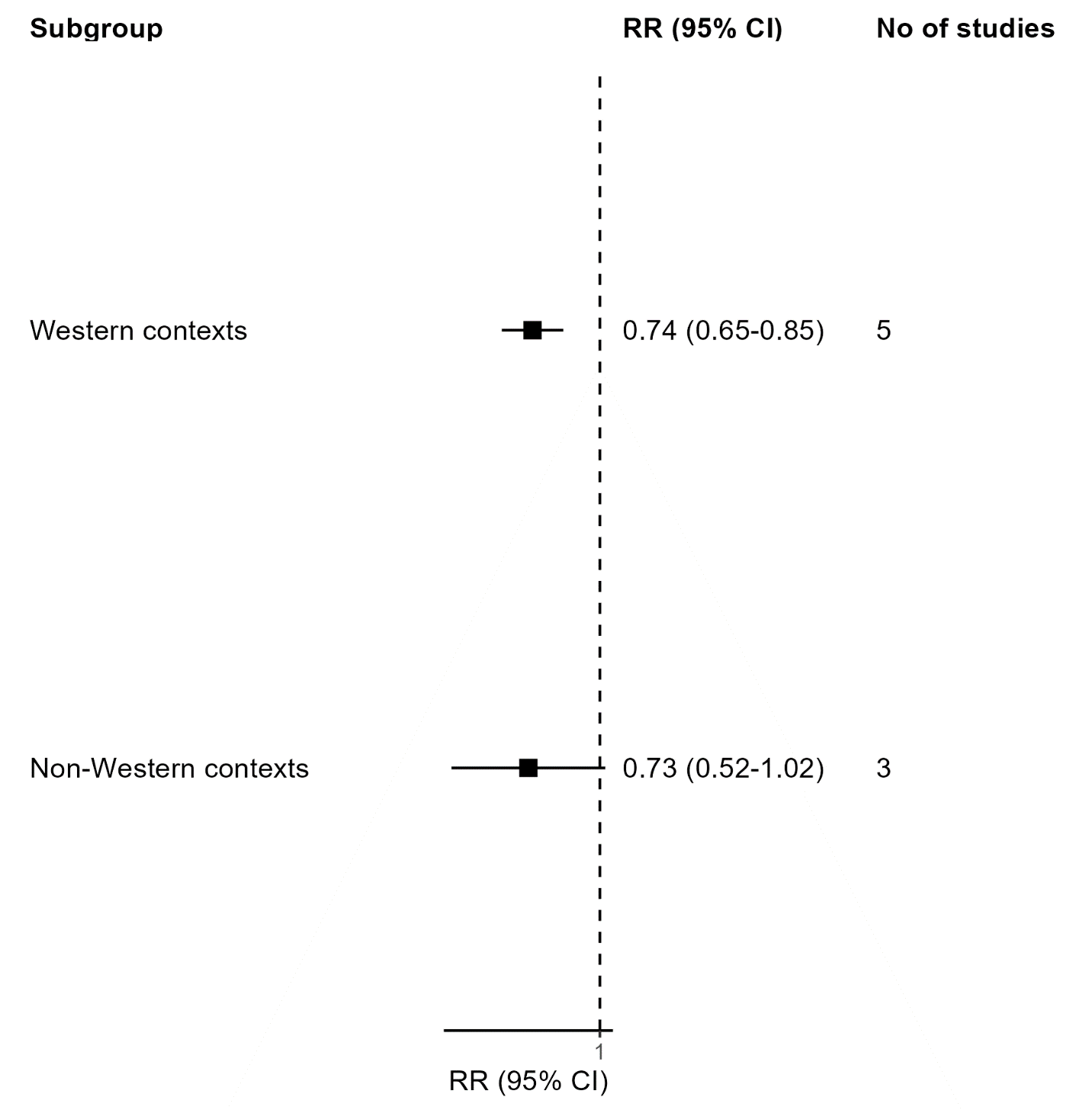


Figure S7: Meta-analyses of unadjusted DBP estimates across subgroups of ≥3 studies or participant subsets


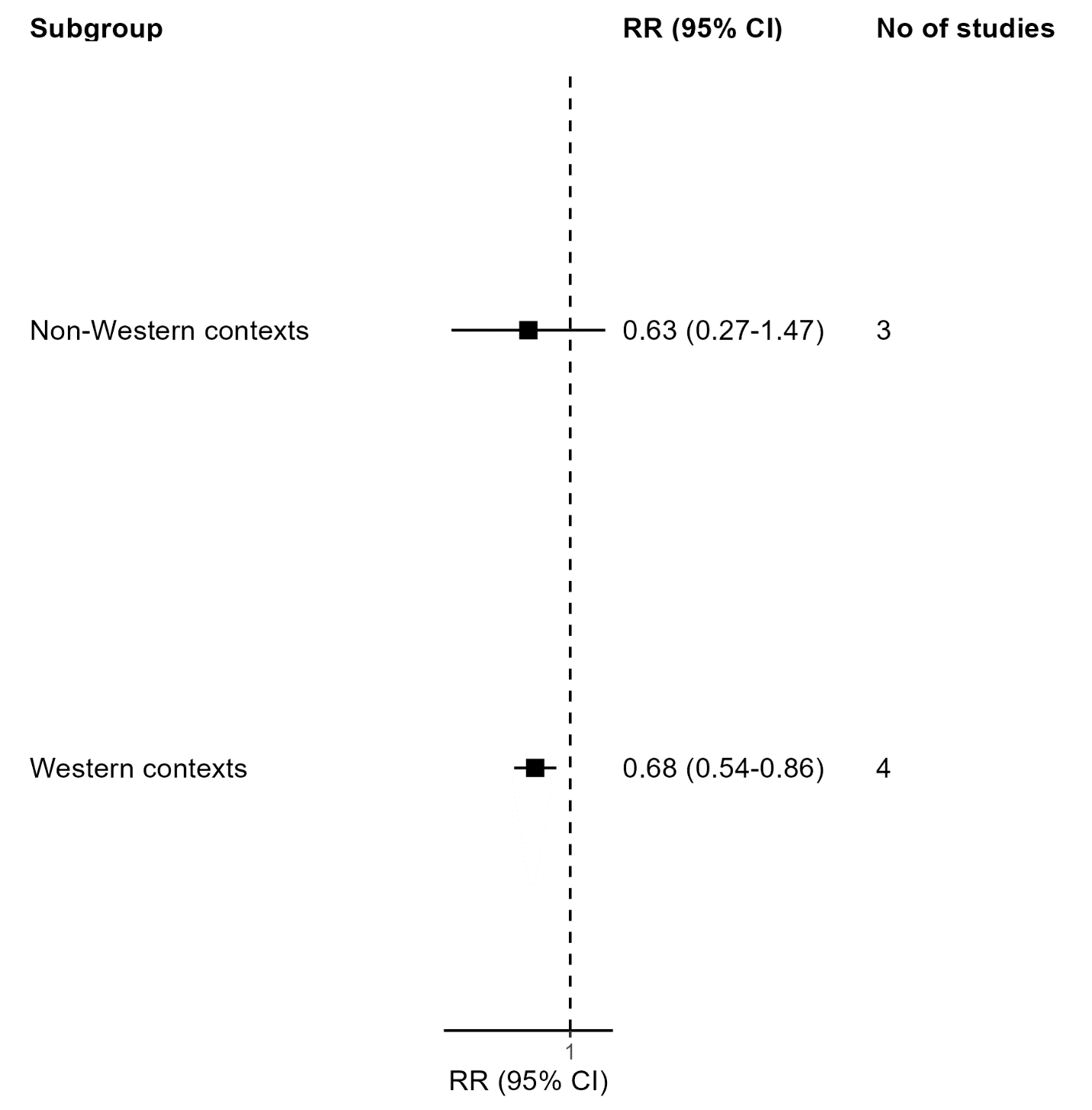


Figure S8: Meta-analyses of unadjusted heart rate estimates across subgroups of ≥3 studies or participant subsets


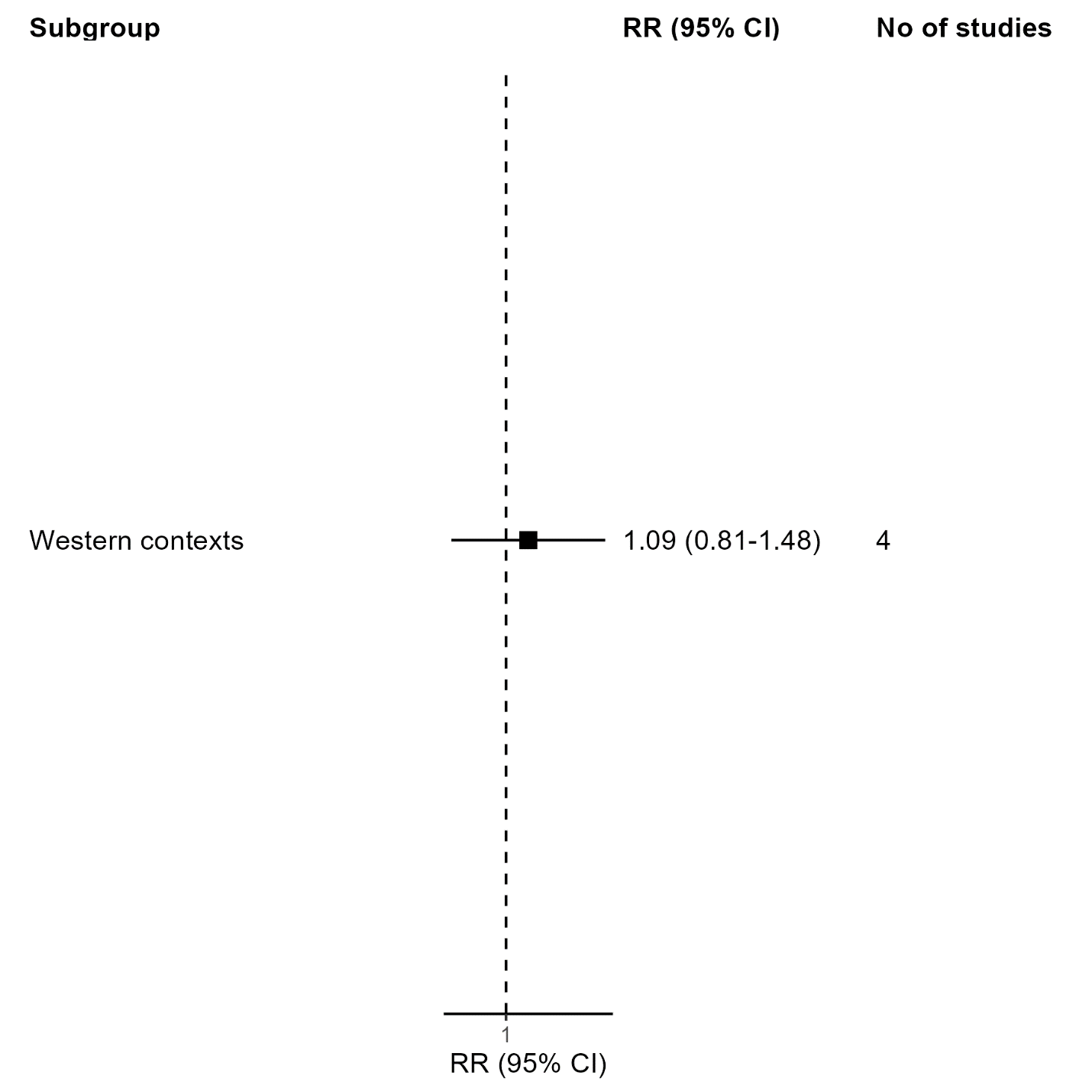


Figure S9: Meta-analyses of unadjusted new (vs returning) donor status estimates across subgroups of ≥3 studies or participant subsets


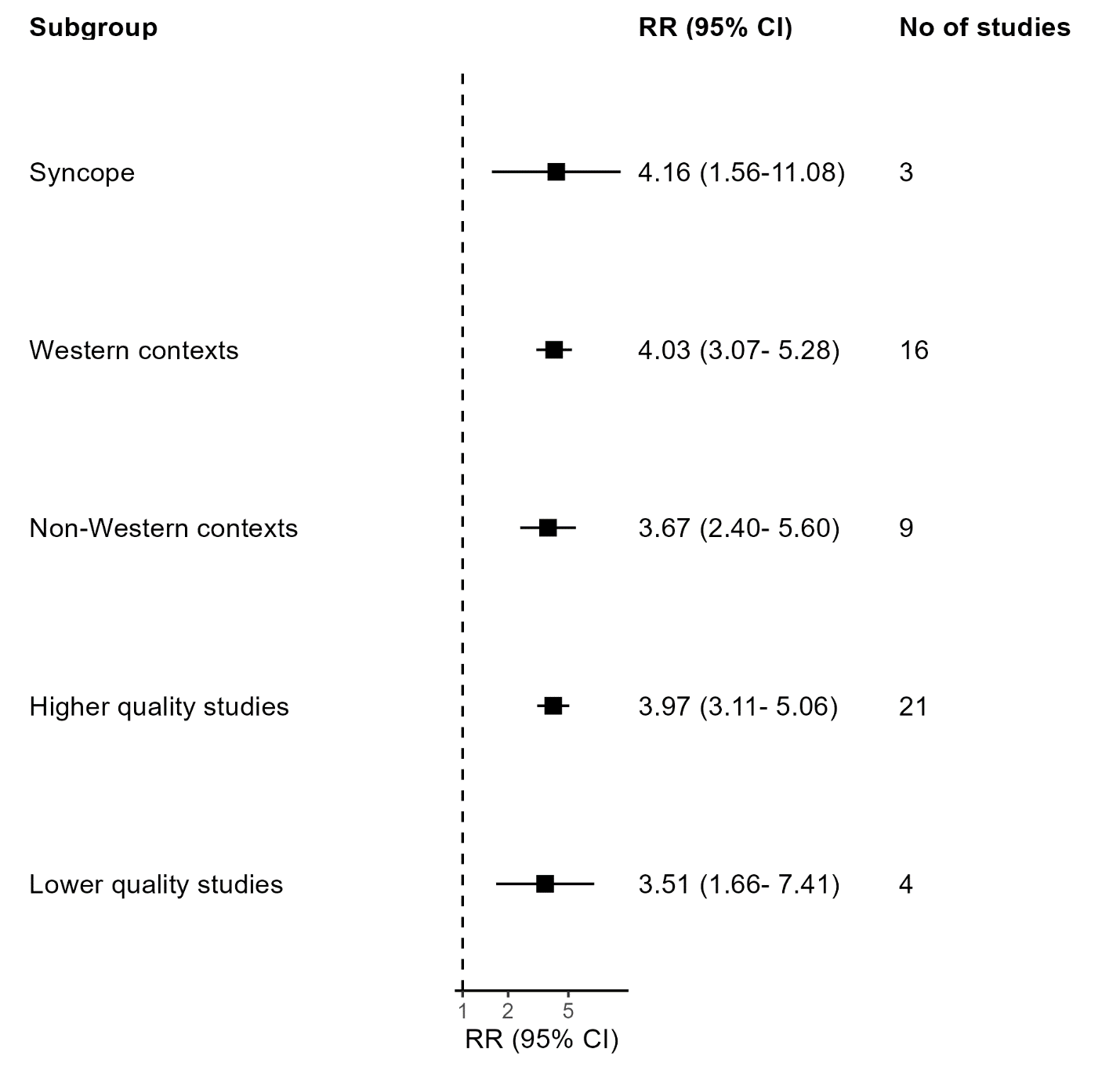


**Appendix S13**. Results from sensitivity analyses

Table S7: Sensitivity analyses for missing risk factor data

| **Risk factor** | **% participants with missing risk factor data** | **Original estimate**  **RR (95% CI)** | **“Best-case” scenario**  **RR (95% CI)** | **“Worst-case” scenario**  **RR (95% CI)** |
| --- | --- | --- | --- | --- |
| **Baseline donor characteristics** | | | | |
| Female (vs male) sex | Rios 2010^11^: <0.01% | 2.10 (1.8, 2.5) | 2.10 (1.81, 2.44) | 2.08 (1.79, 2.43) |
| White  (vs non-white) race/  ethnicity | Bravo 2010^14^: 3.34%  Rios 2010: 2.32% | 1.43 (0.91, 2.25) | 1.62 (1.19, 2.22) | 1.34 (0.77, 2.33) |
| Age  (per one-year increase) | Bravo 2010: <0.01% | 0.95 (0.94, 0.96) | 0.95 (0.94, 0.96) | 0.95 (0.94, 0.96) |
| EBV | Bravo 2010: 1.33%  Rios 2010: 14.4% | 0.61 (0.43, 0.88) | 0.55 (0.35, 0.86) | 0.72 (0.46, 1.13) |
| Weight | Bravo 2010: <0.01%  Rios 2010: 14.3% | 0.57 (0.44, 0.73) | 0.53 (0.31, 0.91) | 0.73 (0.46, 1.16) |
| Height | Bravo 2010: 1.29%  Rios 2010: 14.3% | 0.51 (0.26, 0.98) | 0.15 (0.01, 1.54) | 0.98 (0.13, 7.53) |
| BMI | Bravo 2010: 1.33% | 0.65 (0.40, 1.04) | 0.60 (0.27, 1.29) | 0.74 (0.57, 0.97) |
| **Pre-donation biomarkers** | | | | |
| Heart rate | Bravo 2010: 1.33% | 1.08(0.89, 1.32) | 1.13 (0.92, 1.41) | 1.09 (0.81, 1.46) |
|  |  |  |  |  |
| **Donation-specific factors** | | | | |
| New (vs returning) donor | Inaba 2011^30^: <0.01% | 3.86 (3.09, 4.82) | 3.87 (3.07, 4.89) | 3.84 (3.04, 4.86) |

**Appendix S14**: Assessment of small study effects (including publication bias)

Figure S10: Funnel plots for risk factors examined by ≥10 studies for (1) sex, (2) age, (3) weight, and (4) new (vs returning) donor status

(1) Sex

| Unadjusted  k = 35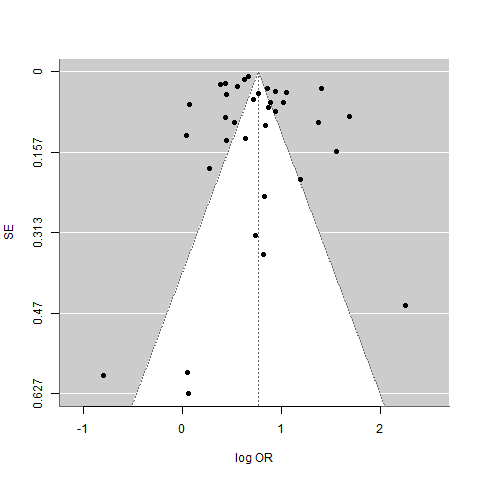 | Adjusted  k = 13  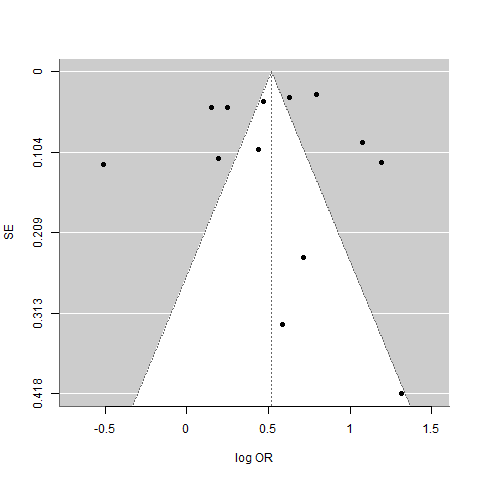 |
| --- | --- |

(2) Age

| Unadjusted  k = 15  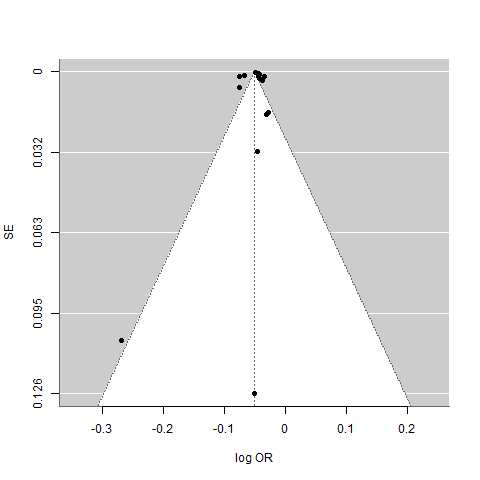 |  |
| --- | --- |

(3) Weight

| Unadjusted  k = 13  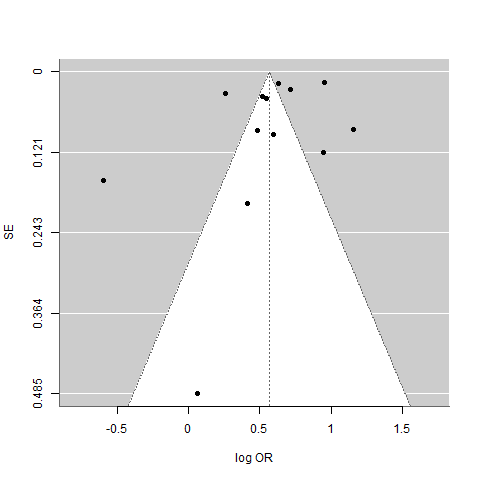 |  |
| --- | --- |

(4) New (vs returning) donor status

| Unadjusted  k = 24  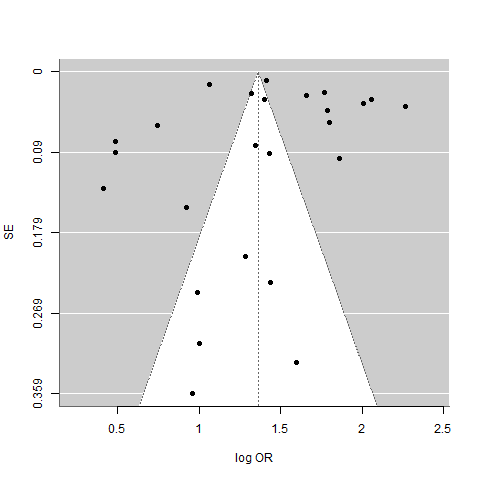 | Adjusted  k = 17  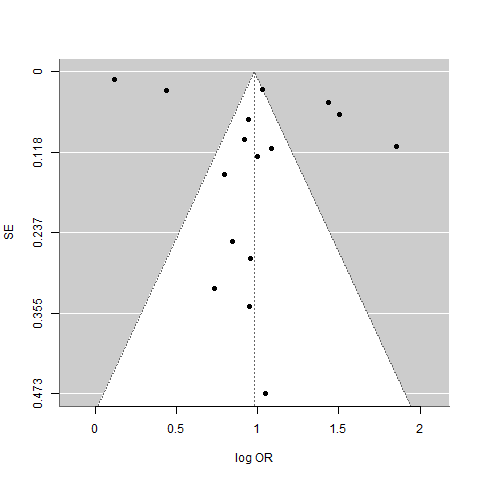 |
| --- | --- |

**References**

1. Standard for Surveillance of Complications Related to Blood Donation. (2014). <https://www.aabb.org/docs/default-source/default-document-library/resources/donor-standard-definitions.pdf>.

2. Faints. (2022). <https://www.transfusionguidelines.org/dsg/wb/guidelines/fa004-faints>.

3. Fainting and feeling faint. (2024). <https://www.lifeblood.com.au/blood/making-your-donation/prepare-and-aftercare/fainting>.

4. Fainting: Advice for donors. (2020). <https://www.scotblood.co.uk/media/2520/faints_web_nov20-final.pdf>.

5. Shi, F., Sun, L., and Kaptoge, S. (2021). Association of beta-2-microglobulin and cardiovascular events and mortality: A systematic review and meta-analysis. Atherosclerosis *320*, 70-78. <https://doi.org/10.1016/j.atherosclerosis.2021.01.018>.

6. Bravo, M., Kamel, H., Custer, B., and Tomasulo, P. (2011). Factors associated with fainting - before, during and after whole blood donation. Vox Sanguinis *101*, 303-312. <https://dx.doi.org/10.1111/j.1423-0410.2011.01494.x>.

7. Hartemink, N., Boshuizen, H.C., Nagelkerke, N.J.D., Jacobs, M.A.M., and van Houwelingen, H.C. (2006). Combining Risk Estimates from Observational Studies with Different Exposure Cutpoints: A Meta-analysis on Body Mass Index and Diabetes Type 2. American Journal of Epidemiology *163*, 1042-1052. 10.1093/aje/kwj141.

8. Stone, C., and Koo, C. (1985). Additive splines in statistics. Proceedings of the Statistical Computing Section, American Statistical Association *27*, 45-48.

9. Orsini, N., Li, R., Wolk, A., Khudyakov, P., and Spiegelman, D. (2012). Meta-analysis for linear and nonlinear dose-response relations: examples, an evaluation of approximations, and software. Am J Epidemiol *175*, 66-73. 10.1093/aje/kwr265.

10. Higgins, J.P., White, I.R., and Wood, A.M. (2008). Imputation methods for missing outcome data in meta-analysis of clinical trials. Clin Trials *5*, 225-239. 10.1177/1740774508091600.

11. Agarwal, R.K., Periyavan, S., Dhanya, R., Parmar, L.G., Sedai, A., Ankita, K., Vaish, A., Sharma, R., and Gowda, P. (2016). Complications related to blood donation: A multicenter study of the prevalence and influencing factors in voluntary blood donation camps in Karnataka, India. Asian journal of transfusion science *10*, 53-58. <https://dx.doi.org/10.4103/0973-6247.165840>.

12. Almutairi, H., Salam, M., Alajlan, A., Wani, F., Al-Shammari, B., and Al-Surimi, K. (2017). Incidence, predictors and severity of adverse events among whole blood donors. PLoS ONE *12*, e0179831. <https://dx.doi.org/10.1371/journal.pone.0179831>.

13. Broadwater, D.R., Krause, K.A., Lynch, D.T., and Kendelhardt, J.D. (2021). Are Bradycardic Donors More Likely to Have Adverse Outcomes During Blood Donation? MILITARY MEDICINE. <https://doi.org/10.1093/milmed/usab416>.

14. Brunson, D.C., Belanger, G.A., Sussmann, H., Fine, A.M., Pandey, S., and Pham, T.D. (2022). Factors associated with first-time and repeat blood donation: Adverse reactions and effects on donor behavior. Transfusion *62*, 1269-1279. <https://dx.doi.org/10.1111/trf.16893>.

15. Burkhardt, T., Dimanski, B., Karl, R., Sievert, U., Karl, A., Hubler, C., Tonn, T., Sopvinik, I., Ertl, H., and Moog, R. (2015). Donor vigilance data of a blood transfusion service: A multicenter analysis. Transfusion and Apheresis Science *53*, 180-184. <https://dx.doi.org/10.1016/j.transci.2015.03.014>.

16. Dunbar, N., Katz, J., and Nambiar, A. (2011). The potential impact of new donor height and weight criteria on young donor eligibility and faint or prefaint reaction rates. Transfusion *51*, 737-741. <https://dx.doi.org/10.1111/j.1537-2995.2010.02918.x>.

17. Eder, A.F., Hillyer, C.D., Dy, B.A., Notari, E.P.t., and Benjamin, R.J. (2008). Adverse reactions to allogeneic whole blood donation by 16- and 17-year-olds. JAMA *299*, 2279-2286. <https://dx.doi.org/10.1001/jama.299.19.2279>.

18. Fraile, N., Fernandez-Cano, E., Ramos, A., Lopez-Aguilar, J.-C., Jimenez-Moreno, M.-E., and Pereira, A. (2021). Vasovagal syncope after blood donation in active duty military personnel of the Spanish Army. Transfusion *61*, 2925-2929. <https://dx.doi.org/10.1111/trf.16603>.

19. France, C.R., Rader, A., and Carlson, B. (2005). Donors who react may not come back: Analysis of repeat donation as a function of phlebotomist ratings of vasovagal reactions. Transfusion and Apheresis Science *33*, 99-106. <https://dx.doi.org/10.1016/j.transci.2005.02.005>.

20. France, C.R., and Hanson, S.A. (2009). Social support attenuates presyncopal reactions to blood donation. Transfusion *49*, 843-850. <https://dx.doi.org/10.1111/j.1537-2995.2008.02057.x>.

21. France, C.R., France, J.L., Himawan, L.K., Stephens, K.Y., Frame-Brown, T.A., Venable, G.A., and Menitove, J.E. (2013). How afraid are you of having blood drawn from your arm? A simple fear question predicts vasovagal reactions without causing them among high school donors. Transfusion *53*, 315-321. <https://dx.doi.org/10.1111/j.1537-2995.2012.03726.x>.

22. France, C.R., France, J.L., Conatser, R., Lux, P., McCullough, J., and Erickson, Y. (2019). Predonation fears identify young donors at risk for vasovagal reactions. Transfusion *59*, 2870‐2875. <https://doi.org/10.1111/trf.15424>.

23. Gillet, P., Rapaille, A., Benoit, A., Ceinos, M., Bertrand, O., de Bouyalsky, I., Govaerts, B., and Lambermont, M. (2015). First-time whole blood donation : A critical step for donor safety and retention on first three donations. Transfusion Clinique et Biologique *22*, 312-317. <https://dx.doi.org/10.1016/j.tracli.2015.09.002>.

24. Goldman, M., Osmond, L., Yi, Q.-L., Cameron-Choi, K., and O'Brien, S.F. (2013). Frequency and risk factors for donor reactions in an anonymous blood donor survey. Transfusion *53*, 1979-1984. <https://dx.doi.org/10.1111/trf.12011>.

25. Goldman, M., Germain, M., Gregoire, Y., Vassallo, R.R., Kamel, H., Bravo, M., Irving, D.O., Di Angelantonio, E., Steele, W.R., and O'Brien, S.F. (2019). Safety of blood donation by individuals over age 70 and their contribution to the blood supply in five developed countries: a BEST Collaborative group study. Transfusion *59*, 1267-1272. <https://dx.doi.org/10.1111/trf.15132>.

26. Goldman, M., Uzicanin, S., Marquis-Boyle, L., and O'Brien, S.F. (2021). Implementation of measures to reduce vasovagal reactions: Donor participation and results. Transfusion *61*, 1764-1771. <https://dx.doi.org/10.1111/trf.16375>.

27. Hasan, I., Arshad, A., Rahim, N.A., and Soo, P.Y. (2020). Vasovagal reaction among whole blood donors in Hospital Pulau Pinang. A statistical-epidemiological study. Asian journal of transfusion science *14*, 28-32. <https://dx.doi.org/10.4103/ajts.AJTS_111_17>.

28. Hashizume, T., Kondo, G., Ishimaru, F., Ikeda, Y., Kagawa, K., Kunii, N., Namba, N., Aoki, K., Sawamura, Y., and Makino, S. (2023). Development and validation of a scoring system to predict vasovagal reaction upon whole-blood donation. Vox Sanguinis *n/a*. <https://doi.org/10.1111/vox.13579>.

29. Ibrahim, N.N., Mohd Noor, N.H., and Zulkafli, Z. (2023). Prevalence and factors associated with vasovagal reaction among whole blood donors in hospital Universiti Sains Malaysia. Transfusion Clinique et Biologique *30*, 238-243. <https://dx.doi.org/10.1016/j.tracli.2023.01.004>.

30. Inaba, S., Takanashi, M., Matsuzaki, K., Ono, Y., Nakajima, K., Shibata, R., Tadokoro, K., Ishikawa, Y., and Kinukawa, N. (2013). Analysis of a questionnaire on adverse reactions to blood donation in Japan. Transfusion and Apheresis Science *48*, 21-34. <https://dx.doi.org/10.1016/j.transci.2012.07.012>.

31. Kuttath, V., Nair, H., and Nair, M. (2021). Role of Predonation Hydration in the Prevention of Postdonation Vasovagal Reactions in first time Blood Donors: a Randomised Controlled Trial. Journal of clinical and diagnostic research *15*, EC47‐EC51. <https://doi.org/10.7860/JCDR/2021/47675.15447>.

32. Majlessi, F., Ghafari, S., Rahimi-Foroushani, A., and Maghsoodlou, M. (2008). Systemic complications and their risk factors among Tehranian blood donor, 2005. Acta Medica Iranica *46*, 253-257.

33. Muller-Steinhardt, M., Muller-Kuller, T., Weiss, C., Menzel, D., Wiesneth, M., Seifried, E., and Kluter, H. (2012). Safety and frequency of whole blood donations from elderly donors. Vox sanguinis *102*, 134-139. <https://dx.doi.org/10.1111/j.1423-0410.2011.01531.x>.

34. Newman, B.H. (2002). Vasovagal reactions in high school students: findings relative to race, risk factor synergism, female sex, and non-high school participants. Transfusion *42*, 1557-1560. <http://dx.doi.org/10.1046/j.1537-2995.2002.00238.x>.

35. Newman, B.H. (2003). Vasovagal reaction rates and body weight: Findings in high- and low-risk populations. Transfusion *43*, 1084-1088. <https://dx.doi.org/10.1046/j.1537-2995.2003.00478.x>.

36. Newman, B.H., Satz, S.L., Janowicz, N.M., and Siegfried, B.A. (2006). Donor reactions in high-school donors: the effects of sex, weight, and collection volume. Transfusion *46*, 284-288. <https://doi.org/10.1111/j.1537-2995.2006.00713.x>.

37. Newman, B., Tommolino, E., Andreozzi, C., Joychan, S., Pocedic, J., and Heringhausen, J. (2007). The effect of a 473-mL (16-oz) water drink on vasovagal donor reaction rates in high-school students. Transfusion *47*, 1524-1533. <https://dx.doi.org/10.1111/j.1537-2995.2007.01293.x>.

38. Nilsson Sojka, B., and Sojka, P. (2003). The blood-donation experience: perceived physical, psychological and social impact of blood donation on the donor. Vox sanguinis *84*, 120-128.

39. Philip, J., Sarkar, R.S., and Jain, N. (2014). A single-centre study of vasovagal reaction in blood donors: Influence of age, sex, donation status, weight, total blood volume and volume of blood collected. Asian journal of transfusion science *8*, 43-46. <https://dx.doi.org/10.4103/0973-6247.126690>.

40. Pisciotto, P., Sataro, P., and Blumberg, N. (1982). Incidence of adverse reactions in blood donors taking antihypertensive medications. Transfusion *22*, 530-531. <https://doi.org/10.1046/j.1537-2995.1982.22683068620.x>.

41. Reiss, R.F., Harkin, R., Lessig, M., and Mascari, J. (2009). Rates of vaso-vagal reactions among first time teenaged whole blood, double red cell, and plateletpheresis donors. Annals of Clinical and Laboratory Science *39*, 138-143.

42. Rios, J.A., Fang, J.Y., Tu, Y.L., Wright, D.J., Spencer, B., Hillyer, C.D., Hillyer, K.L., Eder, A.F., Benjamin, R.J., and Dono, N.R.E. (2010). The potential impact of selective donor deferrals based on estimated blood volume on vasovagal reactions and donor deferral rates. TRANSFUSION *50*, 1265-1275. <https://doi.org/10.1111/j.1537-2995.2009.02578.x>.

43. Sachdev, S., Singh, L., Sharma, R.R., and Marwaha, N. (2017). A Study on the Effect of Pre-donation Salt Loading on Vasovagal Reactions in Young College Going Whole Blood Donors. Indian Journal of Hematology and Blood Transfusion *33*, 592-597. <https://dx.doi.org/10.1007/s12288-017-0787-y>.

44. Schmidt, R.T. (1975). Personality and fainting. Journal of Psychosomatic Research *19*, 21-25. <https://dx.doi.org/10.1016/0022-3999%2875%2990046-X>.

45. Takanashi, M., Odajima, T., Aota, S., Sudoh, M., Yamaga, Y., Ono, Y., Yoshinaga, K., Motoji, T., Matsuzaki, K., Satake, M., et al. (2012). Risk factor analysis of vasovagal reaction from blood donation. Transfusion and Apheresis Science *47*, 319-325. <https://dx.doi.org/10.1016/j.transci.2012.04.002>.

46. Thijsen, A., Masser, B., and Davison, T.E. (2020). Reduced risk of vasovagal reactions in Australian whole blood donors after national implementation of applied muscle tension and water loading. Transfusion *60*, 918-921. <https://dx.doi.org/10.1111/trf.15701>.

47. Tomasulo, P., Kamel, H., Bravo, M., James, R.C., and Custer, B. (2011). Interventions to reduce the vasovagal reaction rate in young whole blood donors. Transfusion *51*, 1511-1521. <https://dx.doi.org/10.1111/j.1537-2995.2011.03074.x>.

48. van den Berg, K., Lam, J., Bruhn, R., Custer, B., and Murphy, E.L. (2012). Water administration and the risk of syncope and presyncope during blood donation: a randomized clinical trial. Transfusion *52*, 2577‐2584. <https://doi.org/10.1111/j.1537-2995.2012.03631.x>.

49. van den Hurk, K., Peffer, K., Habets, K., Atsma, F., Jong, P., van Noord, P.A.H., Veldhuizen, I.J.T., and de Kort, W. (2017). Blood donors' physical characteristics are associated with pre- and post-donation symptoms - Donor InSight. BLOOD TRANSFUSION *15*, 405-412. <https://doi.org/10.2450/2016.0023-16>.

50. Vavic, N., Pagliariccio, A., Bulajic, M., Dinic, R., and Marinozzi, M. (2014). Giving blood donors something to drink before donation can prevent fainting symptoms: Is there a physiological or psychological reason? Transfusion and Apheresis Science *51*, 65-69. <https://dx.doi.org/10.1016/j.transci.2014.03.010>.

51. Veldhuizen, I., Atsma, F., van Dongen, A., and de Kort, W. (2012). Adverse reactions, psychological factors, and their effect on donor retention in men and women. Transfusion *52*, 1871-1879. <https://doi.org/10.1111/j.1537-2995.2011.03551.x>.

52. Wang, H.-H., Chen, P.-M., Lin, C.-L., Jau, R.-C., Hsiao, S.-M., and Ko, J.-L. (2019). Joint effects of risk factors on adverse events associated with adult blood donations. Medicine *98*, e17758. <https://dx.doi.org/10.1097/MD.0000000000017758>.

53. Wiersum-Osselton, J.C., Marijt-van der Kreek, T., Brand, A., Veldhuizen, I., van der Bom, J.G., and de Kort, W. (2014). Risk factors for complications in donors at first and repeat whole blood donation: a cohort study with assessment of the impact on donor return. Blood Transfus *12 Suppl 1*, s28-36. <https://doi.org/10.2450/2013.0262-12>.

54. Wiersum-Osselton, J., Romeijn, B., van den Brekel, E., van Dongen, A., Hermans, F., Bokhorst, A., and Marijt-van der Kreek, T. (2019). Can we prevent vasovagal reactions in young inexperienced whole blood donors? A placebo controlled study comparing effects of a 330 vs 500 mL water drink prior to donation. Transfusion *59*, 555‐565. <https://doi.org/10.1111/trf.15065>.

55. Wiltbank, T.B., Giordano, G.E., Kamel, H., Tomasulo, P., and Custer, B. (2008). Faint and prefaint reactions in whole-blood donors: an analysis of predonation measurements and their predictive value. TRANSFUSION *48*, 1799-1808. <https://doi.org/10.1111/j.1537-2995.2008.01745.x>.

56. Wong, H.K., Lee, C.K., Leung, J.N., Lee, I.Y., and Lin, C.K. (2013). Reduction in vasovagal reaction rate in young first-time blood donors by collecting 350 mL rather than 450 mL. Transfusion *53*, 2763-2765. <https://dx.doi.org/10.1111/trf.12134>.

57. Ditto, B., Adler, P.S., France, C., and France, J. (1995). Family history of hypertension and vasovagal symptoms during blood donation. J Behav Med *18*, 331-340. <https://doi.org/10.1007/bf01857658>.

58. Ditto, B., and France, C.R. (2006). Vasovagal symptoms mediate the relationship between predonation anxiety and subsequent blood donation in female volunteers. Transfusion *46*, 1006-1010. <https://dx.doi.org/10.1111/j.1537-2995.2006.00835.x>.

59. Ditto, B., Gilchrist, P.T., and Holly, C.D. (2012). Fear-related predictors of vasovagal symptoms during blood donation: It’s in the blood. Journal of Behavioral Medicine *35*, 393-399. <https://doi.org/10.1007/s10865-011-9366-0>.

60. Ditto, B., Balegh, S., Gilchrist, P.T., and Holly, C.D. (2012). Relation between perceived blood loss and vasovagal symptoms in blood donors. Clinical Autonomic Research *22*, 113-116. <https://dx.doi.org/10.1007/s10286-011-0147-1>.

61. Ditto, B., Byrne, N., Holly, C., and Balegh, S. (2014). Social contagion of vasovagal reactions in the blood collection clinic: A possible example of mass psychogenic illness. Health Psychology *33*, 639-645. <https://doi.org/10.1037/hea0000053>.

62. Eder, A.F., Notari, E.P.t., and Dodd, R.Y. (2012). Do reactions after whole blood donation predict syncope on return donation? Transfusion *52*, 2570-2576. <https://dx.doi.org/10.1111/j.1537-2995.2012.03666.x>.

63. Ferguson, E. (2001). Intellect and somatic health: Associations with hypochondriacal concerns, perceived threat and fainting. Psychotherapy and Psychosomatics *70*, 319-327. <https://doi.org/10.1159/000056272>.

64. France, C.R., France, J.L., Kowalsky, J.M., Ellis, G.D., Copley, D.M., Geneser, A., Frame-Brown, T., Venable, G., Graham, D., Shipley, P., and Menitove, J.E. (2012). Assessment of donor fear enhances prediction of presyncopal symptoms among volunteer blood donors. Transfusion *52*, 375-380. <https://dx.doi.org/10.1111/j.1537-2995.2011.03294.x>.

65. France, C.R., France, J.L., Frame-Brown, T.A., Venable, G.A., and Menitove, J.E. (2016). Fear of blood draw and total draw time combine to predict vasovagal reactions among whole blood donors. Transfusion *56*, 179-185. <https://dx.doi.org/10.1111/trf.13264>.

66. France, C.R., France, J.L., Kowalsky, J.M., Conatser, R., Duffy, L., Barnofsky, N., Kessler, D., and Shaz, B. (2020). A randomized controlled trial of a tablet-based intervention to address predonation fears among high school donors. Transfusion *60*, 1450‐1453. <https://doi.org/10.1111/trf.15790>.

67. France, C.R., France, J.L., Himawan, L.K., Lux, P., and McCullough, J. (2021). Donation related fears predict vasovagal reactions and donor attrition among high school donors. Transfusion *61*, 102-107. <https://dx.doi.org/10.1111/trf.16099>.

68. Kaloupek, D.G., Scott, J.R., and Khatami, V. (1985). Assessment of coping strategies associated with syncope in blood donors. Journal of Psychosomatic Research *29*, 207-214. <https://doi.org/10.1016/0022-3999(85)90043-1>.

69. Krumholz, A., Ness, P.M., Hauser, W.A., Douglas, D.K., and Gibble, J.W. (1997). Regulations prohibiting blood donation by individuals with seizures or epilepsy are not necessary. Medicine and Law *16*, 339-347.

70. Labus, J.S., France, C.R., and Taylor, B.K. (2000). Vasovagal reactions in volunteer blood donors: Analyzing the predictive power of the Medical Fears Survey. International Journal of Behavioral Medicine *7*, 62-72. <https://doi.org/10.1207/S15327558IJBM0701_5>.

71. Meade, M.A., France, C.R., and Peterson, L.M. (1996). Predicting vasovagal reactions in volunteer blood donors. Journal of Psychosomatic Research *40*, 495-501. <https://doi.org/10.1016/0022-3999(95)00639-7>.

72. Mennitto, S., Harrison, J., Ritz, T., Robillard, P., France, C.R., and Ditto, B. (2019). Respiration and applied tension strategies to reduce vasovagal reactions to blood donation: a randomized controlled trial. Transfusion *59*, 566‐573. <https://doi.org/10.1111/trf.15046>.

73. Mennitto, S., Ritz, T., Robillard, P., France, C.R., and Ditto, B. (2020). Hyperventilation as a Predictor of Blood Donation-Related Vasovagal Symptoms. Psychosomatic medicine *82*, 377‐383. <https://doi.org/10.1097/PSY.0000000000000800>.

74. Mennitto, S., Vachon, D.D., Ritz, T., Robillard, P., France, C.R., and Ditto, B. (2021). Social Contagion of Vasovagal Symptoms in Blood Donors: Interactions With Empathy. Annals of behavioral medicine : a publication of the Society of Behavioral Medicine. <http://dx.doi.org/10.1093/abm/kaab089>.

75. Rudokaite, J., Ertugrul, I.O., Ong, S., Janssen, M.P., and Huis in 't Veld, E. (2023). Predicting Vasovagal Reactions to Needles from Facial Action Units. Journal of Clinical Medicine *12*, 1644. <https://dx.doi.org/10.3390/jcm12041644>.

76. Rudokaite, J., Ong, L.L.S., Onal Ertugrul, I., Janssen, M.P., and Huis In 't Veld, E.M.J. (2023). Predicting vasovagal reactions to needles with anticipatory facial temperature profiles. Scientific reports *13*, 9667. <https://dx.doi.org/10.1038/s41598-023-36207-z>.

77. Stewart, K.R., France, C.R., Rader, A.W., and Stewart, J.C. (2006). Phlebotomist interpersonal skill predicts a reduction in reactions among volunteer blood donors. Transfusion *46*, 1394-1401. <https://dx.doi.org/10.1111/j.1537-2995.2006.00908.x>.

78. Thijsen, A., Masser, B., Davison, T.E., van Dongen, A., and Williams, L.A. (2023). Beyond fear: A longitudinal investigation of emotions and risk of a vasovagal reaction in first-time whole-blood donors. Transfusion *63*, 163-170. <https://dx.doi.org/10.1111/trf.17169>.

79. Viar, M.A., Etzel, E.N., Ciesielski, B.G., and Olatunji, B.O. (2010). Disgust, anxiety, and vasovagal syncope sensations: A comparison of injection-fearful and nonfearful blood donors. Journal of Anxiety Disorders *24*, 941-945. <https://doi.org/10.1016/j.janxdis.2010.06.021>.

80. Vossbeck-Elsebusch, A.N., and Gerlach, A.L. (2012). The relation between disgust-sensitivity, blood-injection-injury fears and vasovagal symptoms in blood donors: Disgust sensitivity cannot explain fainting or blood donation-related symptoms. Journal of Behavior Therapy and Experimental Psychiatry *43*, 607-613. <https://doi.org/10.1016/j.jbtep.2011.08.005>.
